# ﻿Revision of *Acesines* Stål and *Dunnius* Distant, resurrection of *Mycterizon* Breddin (Hemiptera, Heteroptera, Pentatomidae, Pentatominae), and description of a new species from India

**DOI:** 10.3897/zookeys.1148.95629

**Published:** 2023-02-16

**Authors:** Santhamma Salini, R. G. Gracy, Romila Akoijam, Mehaboob K. Rabbani, K. Jacob David, Marcos Roca-Cusachs

**Affiliations:** 1 ICAR–National Bureau of Agricultural Insect Resources, Bangalore, 560024, India ICAR–National Bureau of Agricultural Insect Resources Bangalore India; 2 ICAR-Regional Station, ICAR Complex for NEH Region, Manipur Centre, Lamphelpat, Imphal, 795004, India ICAR-Regional Station Imphal India; 3 IRBio. Institut de Recerca a la Biodiversitat, Universitat de Barcelona, Barcelona, Spain Universitat de Barcelona Barcelona Spain

**Keywords:** *Araductabella*, Axiagastini, lectotype, male genitalia, north-east India

## Abstract

The genera *Acesines* Stål, 1876 and *Dunnius* Distant, 1902 (Hemiptera: Heteroptera: Pentatomidae: Pentatominae) were revised and redescribed with the description of *Dunniusbarpetensis* Salini & Rabbani, **sp. nov.**, based on specimens from Assam and Meghalaya, northeastern states of India. The genus *Mycterizon* Breddin, 1909 is reinstated from *Dunnius*, removed from the tribe Menidini, and redescribed. Consequently, the following new combinations are proposed: *A.sordida* (Kirby, 1891), **comb. nov.**, *Dunniuslaticeps* (Zheng & Liu, 1987), **comb. nov.**, *D.tridentatus* (Xiong & Liu, 1995), **comb. nov.**, and *D.trifasciatus* (Xiong & Liu, 1995), **comb. nov.** A lectotype is designated for *Araductabella* (Distant, 1900a). *Acesinesbambusana* Distant, 1918, and *Mycterizonbellus***stat. rev.** are redescribed based on both male and female genitalia and *Dunniusfulvescens* (Dallas, 1851) is redescribed based on female genitalia.

## ﻿Introduction

The genus *Acesines* was first proposed by Stål (1876) to accommodate *Acesinesbreviceps* Stål, 1876 which he placed in the “*Axiagastus* et affinia” group. The genus currently comprises five species, namely *Acesinesbambusana* Distant, 1918, *A.breviceps*, *A.laticeps* Zheng & Liu, 1987, *A.tridentatus* Xiong & Liu, 1995, and *A.trifasciatus* Xiong & Liu, 1995, with their distribution restricted to Asia. Of these, *A.bambusana* and *A.breviceps* are the only species so far recorded from India (Stål 1876; Atkinson 1888; Distant 1918).

Stål (1876) defined *Acesines* mainly with the following set of diagnostic characters: short head, narrowly reflexed anterolateral margins of pronotum, labium reaching metasternum, mesosteral carina gradually thickened posteriorly, metasternal carina elevated and hexagonal; basal abdominal segment with prominent tubercle touching the metasternal carina. Atkinson (1888) redescribed *Acesines* and *A.breviceps* based on external morphology and followed Stål (1876) in placing the genus in the division Axiagastaria (= Axiagastini). However, Distant (1902) did not recognise the Axiagastini as a valid tribe and considered *Acesines* to belong in the Menidaria (= Menidini). In addition, Distant (1902) also proposed the genus *Dunnius* in Menidaria to accommodate a few closely related species, namely *Dunniusfulvescens* (Dallas, 1851), *D.sordida* (Kirby, 1891), and *D.bellus* (Distant, 1900a). *D.bellus* was originally described in the genus *Araducta*Walker (1867) by Distant (1900a); later, Distant (1902) moved *Araductabella* to *Dunnius* for unknown reasons. However, by recognising its unique diagnostics such as a broad and much shorter head, markedly sinuate lateral margins of head, antennomere I nearly reaching apex of head, short labium, broad apex of scutellum, short peritreme freely protruding from metapleuron, and ventrite III flat but with short, convex region medially, Breddin (1909) proposed *Mycterizon* to accommodate *A.bella*. Breddin (1909) also commented that *Mycterizon* had nothing to do with *Dunnius* or *Hippota* Bergroth but was instead closely related to *Antestia* Stål. Distant (1918) subsequently synonymised *Mycterizon* with *Dunnius* again for unknown reasons. Zheng and Liu (1987) added another species, *Dunniusminor* from Yunnan, China. All the four species of *Dunnius* except *D.minor* are recorded from India (Distant 1900b, 1902; Chatterjee 1934) although the record of *D.sordida* by Chatterjee (1934) needs confirmation.

Two specimens resembling *Acesines* were collected as part of the insect collection surveys, which were undertaken to the northeastern region of India, led the authors to investigate the taxonomic position of various species currently accommodated in genera *Acesines* and *Dunnius*. Hence this paper aims at revising these two genera, resurrecting the genus *Mycterizon* for *D.bellus*, describing a new species of *Dunnius*, redescribing *D.fulvescens* (the type species of *Dunnius*) based on female genitalia, and redescribing *A.bambusana* based on male and female genitalia. *Dunniussordida* is transferred from *Dunnius* to *Acesines* and *A.laticeps*, *A.tridentatus*, and *A.trifasciatus* are transferred from *Acesines* to *Dunnius*. Additionally, taxonomic notes on *Acesinessordida* (Kirby, 1891), comb. nov. *Dunniuslaticeps* (Zheng & Liu, 1987), comb. nov., *Dunniustridentatus* (Xiong & Liu, 1995), comb. nov., *Dunniustrifasciatus* (Xiong & Liu, 1995), comb. nov., and *A.breviceps* are provided. As the types of the aforementioned species were not available for study, detailed redescriptions are not included.

## ﻿Materials and methods

All the specimens examined for the present study are deposited in the National Insect Museum (**NIM**), Indian Council of Agricultural Research-National Bureau of Agricultural Insect Resources, Bangalore, Karnataka, India (ICAR-NBAIR). Observations, dissections, and illustrations of male and female genitalia and general morphology were made using the stereozoom microscope, Nikon SMZ 745 (model C-FIR). Dissection and inflation of male genitalia followed Salini (2016). The female genitalia was dissected after boiling the complete abdomen in hot water for ca. 10–15 min with 10% potassium hydroxide (KOH). The internal contents were cleared after thoroughly washing it in distilled water two or three times, and the terminalia and spermatheca were detached from abdominal ventrites with the help of fine forceps. One female and one male each of the new species were examined for the present work. Photographs and measurements were made using DFC 420 camera mounted on Leica M205A Stereomicroscope and LEICA DMC 4500 with Leica Automontage Software (LAS). All measurements are given in millimetres and presented as median, with minimum and maximum values given between parentheses. The following dimensions were measured in dorsal view: body length (from apex of mandibular plates to apex of membrane or apex of tergite VIII), head length (from apex of mandibular plates to anterior margin of pronotum), head width (width of head across compound eyes), interocular width (between inner margins of compound eyes), length of each antennomere, length of each labiomere, pronotum length (medially, from anterior to posterior margin of pronotum), pronotum width (maximum width between humeri), scutellum length (medially from base to apex) and scutellum width (maximum width between basal angles).

The type images of *A.breviceps* were obtained from Gunvi Lindberg, Swedish Museum of Natural History (**SMNH**), Stockholm, Sweden; *D.sordida* and *D.bellus* from Mick Webb, Natural History Museum (**NHM**), UK, London, and the dorsal habitus images of types of *D.fulvescens* was obtained from Petr Kment, National Museum (**NMP**), Prague, Czech Republic. The authors have seen neither the types nor the type images of *A.laticeps*, *A.tridentatus*, and *A.trifasciatus* as attempts to obtain those were not successful. Therefore, the transfer of species, viz., *A.laticeps*, *A.tridentatus*, and *A.trifasciatus* from *Acesines* to *Dunnius* was decided on consultation with the literature including the original descriptions of those species by Zheng and Liu (1987), Xiong and Liu (1995), and Fan (2011). The transfer of *D.sordida* to *Acesines* was based on the type images of *D.sordida* and in the literature (Kirby 1891; Distant 1900a, 1902). *Dunniusfulvescens* is redescribed based on the single available female specimen from Arunachal Pradesh. Detailed redescriptions of *A.sordida*, *A.breviceps*, *D.laticeps*, *D.tridentatus*, and *D.trifasciatus* are not included as neither the types nor the identified material of these species was available for the study.

Terminology of general morphology follows Tsai et al. (2011) and Salini and Kment (2021), specialised terms follow the following papers: antennomeres (Zrzavý 1990) and superior processes of dorsal rim (Roell et al. 2020). Terminology of male and female genitalia follows Tsai et al. (2011) and those associated with the metathoracic scent glands follow Kment and Vilímová (2010).

## ﻿Taxonomic account

### ﻿ Acesines

Taxon classificationAnimaliaHemipteraPentatomidae

Stål, 1876

935DA215-7425-5D65-9099-4D0239A9EF95

Acesines Stål, 1876: 65 (key to genera), 94 (description of type species). Type species: Acesinesbreviceps Stål, 1876: 94–95, by monotypy.
Acesines : Atkinson (1888): 131–132 (redescription); Lethierry and Severin (1893): 171 (catalogue); Distant (1902): 225, 231 (key to genera, distribution, redescription); Kirkaldy (1909): 127 (catalogue); Rider (2006): 259 (Palearctic catalogue); Fan (2011): 210 (species transfer); Salini and Viraktamath (2015): 9 (key to genera), 16 (checklist); Rider et al. (2018): 67, 74, 75, 89 (comments on tribal placement).

#### Redescription.

***Colouration*.** Body pale brown with mosaic of ochraceous irregular patches; antennae pale; membrane pale brown, translucent; head and thorax (ventral side), ventral side of abdomen medially, pale yellowish; legs (except apical 1/2 of claws, black) and labium (except apical 1/2 of segment IV of labium, black) concolourous to abdomen.

***Integument and vestiture*.** Body above covered with coarse, dark brown punctures; head (ventrally) and thoracic pleura with coarse, brown punctures; abdominal sternites with fine, brown punctures. Body glabrous, except antennae and legs moderately pilose. Male genitalia with ventral rim including the posterolateral lobes of the genital capsule, possessing short, golden setae. Female genitalia with valvifers VIII and IX, laterotergites VIII and IX and abdominal segment X provided with short setae.

***Structure*.** Head (Fig. 4) above flat, sloping downwards, much shorter than wide, lateral margins narrowly reflexed, slightly concave in front of compound eyes. Mandibular plates much wider, but as long as clypeus, not meeting in front of clypeus. Clypeus slightly narrowed towards apex. Compound eyes moderately large, rounded, protruding out of the head outline in most of their width. Ocelli small, situated posteriorly near to anterior margin of pronotum. Antenniferous tubercles small or indistinct. Antennae with five antennomeres, slender, antennomeres from shortest to longest: I<II_a_<II_b_<IV<III; antennomere I cylindrical, shortest and stoutest, remaining antennomeres cylindrical and slender. Bucculae with anterior apex slightly angular, shorter than labial segment I. Labium reaching metacoxae.

***Pronotum*** (Fig. 5). Anterior margin not collar-like, rather thin, not possessing the median concavity to accommodate posterior margin of head; anterolateral angles with minute laterally directed tooth. Anterolateral margin obliquely straight, narrowly reflexed; posterior margin nearly straight, posterolateral angles rounded; humeri rounded. Disc of pronotum strongly convex with anterior 2/3 sloping downwards.

***Scutellum*.** Subtriangular, longer than broad at base; scutellar disc with basal 1/2 gibbous; posteriorly flat. Apex of scutellum narrowly rounded.

***Hemelytra*.** Corium with anterodistal angles rounded, extending well beyond apex of scutellum. Membrane translucent with five or six simple veins, without reticulate venation.

***Thoracic pleuron and sternum*.** Pro- and meso-sternum with median longitudinal carina abruptly narrowed anteriorly, not extending beyond procoxae; posteriorly in contact with nearly hexagonal metasternal carina; metasternal carina posteriorly hollowed out or grooved (Fig. 7) to accommodate apex of basal abdominal tubercle. External scent efferent system (Fig. 3) with peritreme well developed into spout-like shape, reaching 1/3 of metapleural width.

***Legs*.** All femora unarmed, cylindrical, rounded in cross-section. All tibiae triangular in cross-section, their outer surface sulcate. All tarsi with segment II shortest, dorsally regularly rounded.

***Pregenital abdomen*.** Connexivum slightly exposed. Posterolateral angles of abdominal ventrites angulate. Abdominal venter slightly convex medially, neither grooved nor keeled; ventrite III medially with prominent tubercle (Fig. 7), apex of which is lodged in groove at posterior apex of metasternal carina.

***Male genitalia*.** Genital capsule (Figs 8, 10–20) nearly quadrangular with posterolateral lobes (= caudal lobes) well developed. Dorsal rim wide and excavated nearly as deep as ventral rim with wavy outline. Ventral rim medially widely excavated concave. A pair each of superior processes of dorsal rim (Figs 15, 16) and parameres present (Figs 13, 14). Parameral crown forked at apex, short neck and plate-like apodeme (ad). *Phallus*. Articulatory apparatus with 1+1 conical capitate processes. Phallotheca twice as wide as long, dorsal region bulbous a pair of large, membranous, dorsal conjunctival processes (dcp), bilobed apically and distal end hooked. Processes of aedeagus (pa) fused into tongue-like structure. Aedeagus short, tubular with oblique phallotreme.

***Female genitalia*** (Figs 21–23). Valvifers VIII (vf VIII) transverse, broad and roughly subquadrangular; valvifers IX (vf IX) single, transverse, large subtrapezoidal sclerite; laterotergites IX (lt IX) oblique, elongate, broadly rounded caudal apex; laterotergites VIII (lt VIII) subtriangular; caudal margin of laterotergite VIII medially with short but wide triangular projection. A pair of ring sclerites (rs) elongate oval. Spermathecal dilation long (Fig. 23), regularly, obliquely fluted at distal end and with another short dilation proximally; both dilations connected by a narrow median region; apical receptacle (ar) elongate, constricted at middle, forming a double globular shape with four ductules.

#### Differential diagnosis and remarks.

*Acesines*, based on morphological features, is closely related to *Dunnius* and has been treated as congeneric (Rider et al. 2018). Based on the specimens examined under these two genera, *Acesines* can be easily differentiated by the hollowed out or grooved (or notched) posterior apex of the metasternal carina (Fig. 7) which aid to accommodate the apex of basal abdominal tubercle. Moreover, the posterior apex of metasternal carina is not grooved or hollowed out, rather cruciform (Fig. 56) in the case of *Dunnius*, which was mentioned in the original description by Distant (1902). In this context, the species currently placed in *Acesines* need re-examination. *Acesines* currently accommodates five species, three of which are known only from China, one is only known from India, and the last is known from both India and Thailand (Rider et al. 2018). Based on the above-mentioned diagnostic with the similarity of male genitalia characters (shape of paramere and shape of caudal lobes of genital capsule), the species namely *A.laticeps*, *A.tridentatus*, and *A.trifasciatus* are removed from this genus (see differential diagnosis and remarks under *Dunnius*). This action results in the exclusion of *Acesines* from the Chinese fauna. In addition, *D.sordida* needs to be transferred from *Dunnius* to *Acesines*, based on the notched posterior apex of metasternal carina, which is evident in the type images (Fig. 35; images were provided by courtesy of M Webb, NHM, UK). Therefore, this genus extends its distribution to Sri Lanka and presently contains three species: *A.bambusana*, *A.breviceps*, and *A.sordida*.

#### Etymology.

Neither the etymology nor the gender of the name *Acesines* were explicitly given in the original description. The name *Acesines* is probably borrowed from ancient Greek (meaning a tributary river of Indus), which is masculine according to the Article 30.1.4.2 of ICZN (1999).

### ﻿ Acesines bambusana

Taxon classificationAnimaliaHemipteraPentatomidae

Distant, 1918

4682B253-F4FB-58D5-939D-046512CC364E

Figs 1–6 , 7–12 , 13–19 , 20–25

Acesines bambusana Distant, 1918: 144 (original description). Holotype, India, Karnataka (NHM).
Acesines bambusana : Usman and Puttarudriah (1955) (list); Salini and Viraktamath (2015): 16 (checklist), 42 fig. 29 (illustration of dorsal habitus), 52, fig. 92 (illustration of head), 57, fig. 124 (illustration of head and pronotum), 65, fig. 167 (illustration of external scent efferent system), 72, fig. 210 (illustration of ventral habitus); Salini (2019): 137 (host).

#### Type locality.

*Acesinesbambusana*. India, Karnataka, south India, Chikkaballapura (= Chikkaballapur), T. V. Campbell Coll., 13.4355°N, 77.7315°E (Fig. 24).

#### Type material.

*Acesinesbambusana*. Not examined, only image of type was seen. The original description seems to be based on more than one specimen as the length of the examined specimens is given as a range. The type is deposited in NHM, London, UK. The image of the syntype (Fig. 25) is provided courtesy of P Kment.

Many of the Indian types described by Distant (1918) came from the collection of T.V. Campbell and were acquired by the Natural History Museum, London (ex-BMNH) as part of Distant’s collection (Shobharani et al. 2018). In the case of *A.bambusana* this also occurs, there are 19 specimens in the NHM, which were obtained by the museum after its original description by Distant (1918). However, one syntype has a holotype label, which has an unknown acquisition date as it lacks a registration label. Hence this syntype specimen might have come to the NHM at a separate time but before its description and therefore it could be considered as a syntype. Moreover, this specimen is a pinned one, unlike the remaining specimens that are card mounted. The registration numbers on T. V. Campbell’s collection indicate when the material was donated to the museum and any material obtained after the original description may or may not be type material. Therefore, the 18 specimens, excluding the syntype having the holotype label, are considered to be specimens with uncertain type status, which are provided with handwritten information such as C. B. 7.15; C. B. 8.15 and C. B. 9.15; these are abbreviations for Chikkaballapura and probably the date of collection (M Webb, pers. comm.).

**Figures 1–6.**
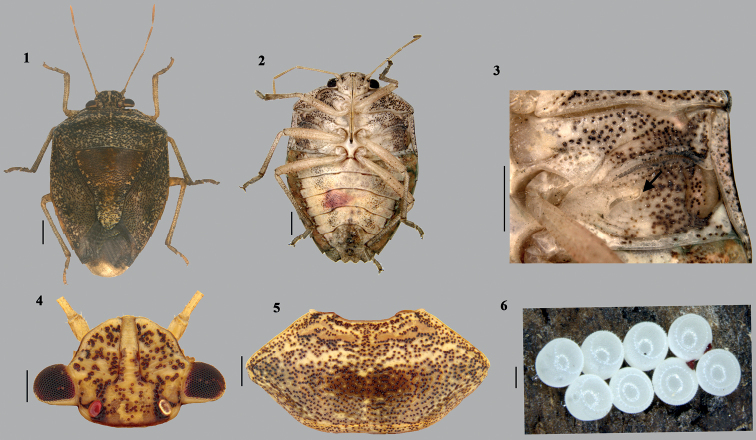
*Acesinesbambusana* Distant **1** habitus (dorsal) **2** habitus (ventral) **3** external scent efferent system (arrow showing the peritreme) **4** head (dorsal) **5** pronotum **6** eggs in clusters. Scale bars: 1 mm (**1–3**); 0.5 mm (**4–6**).

#### Material examined.

**India: Karnataka**: 1♀, Shimoga, 06.x.2008, Umeshkumar; 1♂, NBAII (NIM); 1♀, Shimoga, 05.x.2008, Ex. light trap (sodium vapour lamp), Yeshwanth, H. M., NBAII (NIM); 1♀, 1♂, Shimoga, 06.x.2008, Ex. light trap (sodium vapour lamp), Umeshkumar, NBAII (NIM); 1♀, Kandali, Hassan [12°58.810'N, 76°02.513'E], 11.xi.2008, Yeshwanth, NBAII (NIM); 1♀, Forestry College Sirsi, 08.xii.2007, Ex. light trap (sodium vapour lamp), Umeshkumar, I. S., NBAII (NIM); 1♀, Hesaraghatta, 1.xii.2008, Umeshkumar (NIM); 1♂, 1♀, B.R. Hills, Chamarajanagar, 22.viii.2007, Ex. light trap (sodium vapour lamp), Yadava Babu R.K., NBAII (NIM); 1♂, Bengaluru, Attur, 19.ii.2018, Prabhu, G. (NIM); 1♀, B.R. Hills, Chamarajanagar, 22.viii.2007 Ex. light trap (sodium vapour lamp), Naveen Kumar, N., NBAII (NIM); 1♂, Shimoga, 06.x.2008, Ex. light trap (sodium vapour lamp), Yeshwanth, H.M., NBAII (NIM); 1♀, Bangalore, Hebbal, 04.ix.2013, Salini, S. (NIM); **Tamil Nadu**: 1♀, Annamalai Univ. Campus, 11°23'N, 79°43'E, 4.ii.2010, Umeshkumar, NBAII (NIM).

#### Redescription.

***Colouration*** (Figs 1, 2). Head above, pronotum, scutellum (paler basally), and hemelytra pale brown with mosaic of ochraceous irregular patches; apex of scutellum with outer margin, black; circumference of body (head, pronotum, anterolateral margin of hemelytra and abdomen) and minute spinous projection at posterolateral end of abdominal sternites, black; antennae pale; membrane pale brown, translucent; head and thorax (ventral side), ventral side of abdomen medially, pale yellowish and outer narrow marginal area pale brown; legs (apical 1/2 of claws black) and labium (apical 1/2 of segment IV of labium concolourous to abdomen.

**Figures 7–12.**
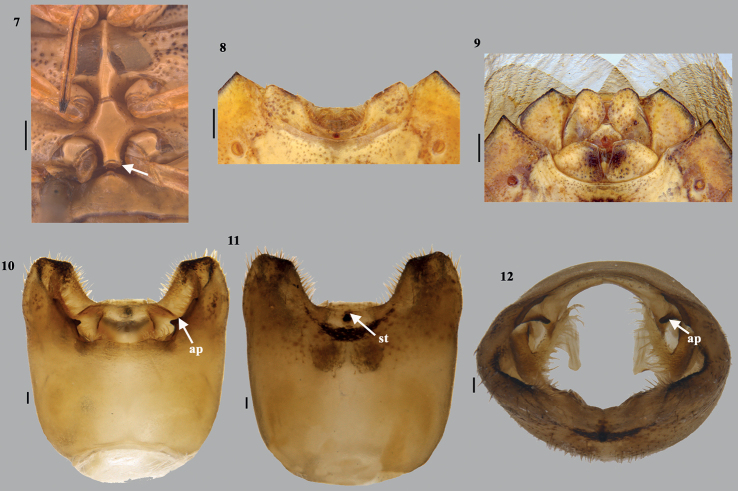
*Acesinesbambusana* Distant **7** metasternal carina (arrow showing the hollowed out or grooved metasternal carina) **8** posterior apex of male abdomen **9** posterior apex of female abdomen **10** genital capsule (dorsal) **11** genital capsule (ventral) **12** genital capsule (caudal). Scale bars: 0.5 mm (**7–9**); 0.1 mm (**10–12**). Abbreviations: ap–angular process; st–short sclerotised tooth.

**Figures 13–19.**
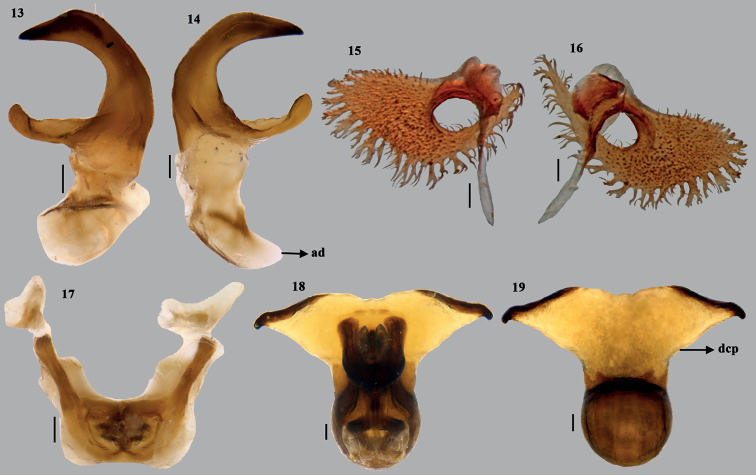
*Acesinesbambusana* Distant **13, 14** parameres (different planes) **15, 16** superior processes of dorsal rim (different planes)**17** articulatory apparatus **18** phallus (ventral) **19** phallus (dorsal). Scale bars: 0.1 mm. Abbreviations: ad– apodeme; dcp– dorsal conjunctival processes.

**Figures 20–25.**
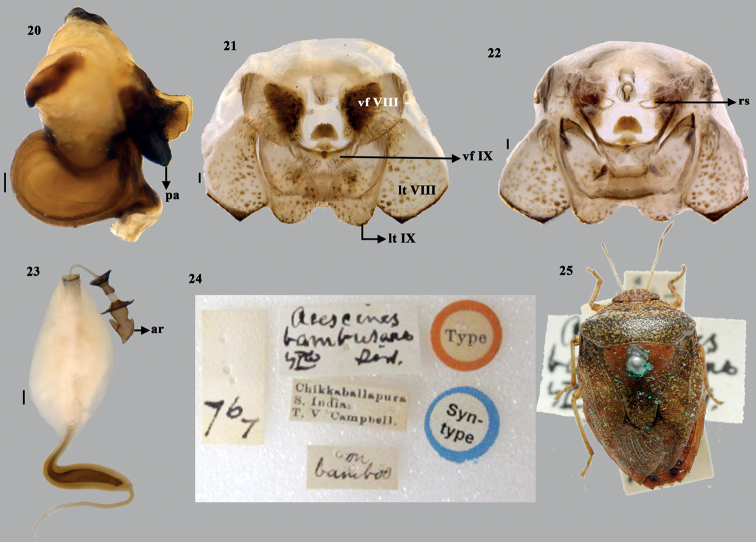
*Acesinesbambusana* Distant **20** phallus (lateral) **21** terminalia (dorsal) **22** terminalia (ventral) **23** spermatheca **24** syntype label Mick Webb, NHM, London, UK **25** syntype (dorsal habitus), courtesy P. Kment. Abbreviations: ar–apical receptacle; pa–processes of aedeagus; vf VIII–valvifers VIII; vf IX–valvifers IX; lt VIII–laterotergite VIII; lt IX–laterotergite IX. Scale bars: 0.1 mm (**20–23**).

***Integument and vestiture*.** Body above covered with coarse, dark brown punctures; head (ventrally) and thoracic pleura with coarse, brown punctures; abdominal sternites (lateral region surrounding the subrounded fields enclosing the spiracle) with fine, brown punctures. Body glabrous except antennae and legs moderately pilose. Male genitalia with ventral rim including the posterolateral lobes of the genital capsule, possessing short, golden setae. Female genitalia with valvifers VIII and IX, laterotergites VIII and IX, and abdominal segment X provided with short setae.

***Structure*.** See the generic redescription.

***Male genitalia*.** Genital capsule (Figs 8, 10–20) nearly quadrangular with posterolateral lobes (= caudal lobes) well developed. Dorsal rim wide and excavated nearly as deep as ventral rim with wavy outline; 1+1 triangular, blunt projection on inner side adjacent to the dorso- lateral wall of the dorsal rim. A short, sclerotised tooth (st) at middle and anterior to dorsal rim. Ventral rim medially wide U-shaped with a narrow median emargination, infoldings of ventral rim well developed with a 1+1 angulate process (ap) sublaterally. A pair each of superior processes of dorsal rim and parameres present. Superior processes of dorsal rim slightly sclerotised, lamellate with serrated outer margin. Parameral crown forked at apex, short neck and plate-like apodeme (ad). ***Articulatory apparatus*.** Support bridge complex excavated U-shaped on dorsal side, with two broad arm-like structures, for which the cone-like capitate processes attached (Fig. 17) with dorsal connectives. ***Phallus*.** Phallotheca twice as wide as long, dorsal region bulbous (Fig. 20) and ventral margin slightly concave towards proximal end (in lateral view); a pair of large, membranous, dorsal conjunctival processes (dcp), bilobed apically; caudal region of each lobe of dorsal conjunctival processes with elongate, narrow strip ending in the hooked sclerotised tips. Dorsal conjunctival process enclosing the base of sclerotised processes of aedeagus on its dorsal and lateral sides. Processes of aedeagus (pa) fused into tongue-like structure, contralateral counterparts medially forming as flap-like lobes (positioned in vertical plane to the plane of processes of aedeagus), slightly longer than aedeagus and enclosing it laterally, extreme apices fused medially, forming a median fusion line, but with shallow U-notch at the apex. Aedeagus short, tubular with oblique phallotreme.

***Female genitalia*.** (Figs 21–23). Valvifers VIII transverse, broad and roughly subquadrangular, with medial margins slightly concave posteriorly; inner posterolateral angles angulate; posterior margin straight; valvifers IX single, transverse, large subtrapezoidal sclerite; laterotergites IX oblique, elongate, broadly rounded caudal apex; laterotergites VIII subtriangular connected each other with a narrow, straight region; caudal margin of laterotergite VIII medially with short but wide triangular projection. A pair of ring sclerites (rs) elongate oval. ***Spermatheca*.** Spermathecal dilation long, regularly, obliquely fluted at distal end and with another short dilation proximally; both dilations connected by a narrow median region; distal spermathecal duct narrow and longer than proximal one; proximal spermathecal duct narrow and tubular; proximal flange shorter than distal flange; apical receptacle (ar) elongate, constricted at middle, forming a double globular shape with four ductules, of which one elongate, facing towards distal rim and not reaching distal rim.

#### Measurements

**(mm).** Males (*n* = 5) as median (minimum–maximum). Body length 12.15 (10.13–15.85); head: length 1.71 (1.57–1.97), width (including eyes) 2.99 (2.61–3.51), interocular width 1.65 (1.51–1.88); lengths of antennomeres: I –0.55 (0.48–0.64), II – 1.16 (0.96–1.41), III – 1.40 (1.21–1.77), IV – 1.64 (1.43–1.97), V – 1.38 (1.19–1.54); lengths of labiomeres: I– 0.80 (0.61–1.03), II– 1.35 (1.10–1.66), III– 1.07 (1.00–1.18), IV– 0.64 (0.55–0.77); pronotum: length 2.99 (2.45–3.88), width (including humeri) 6.99 (6.12–8.31); scutellum: length 4.87 (4.13–5.99), width (at basal angles) 4.60 (4.00–5.55).

Females (*n* = 5) as median (minimum–maximum). Body length 11.54 (11.08–11.94); head: length 1.63 (1.56–1.66), width (including eyes) 2.98 (2.89–3.03), interocular width 1.65 (1.60–1.75); lengths of antennomeres: I –0.45 (0.42–0.47), II – 1.07 (1.01–1.13), III – 1.30 (1.27–1.32), IV – 1.53 (1.41–1.60), V –1.21 (1.18–1.24); length of labiomeres: I –0.67 (0.63–0.71), II –1.25 (1.13–1.33), III – 1.02 (0.98–1.13), IV – 0.62 (0.59–0.65); pronotum: length 3.12 (2.83–3.33), width (including humeri) 6.92 (6.77–7.03); scutellum: length 4.89 (4.73–5.03), width (at basal angles) 4.55 (4.35–4.66).

#### Differential diagnosis.

This species can be differentiated by the peculiar C-shaped crown of paramere, expanded dorsal conjunctival processes with hooked distal apex and the external female genitalia characters such as the short but wide triangular projection at middle the caudal margin of laterotergites VIII and the sclerotised rod-like structure expanded to an elongate, oval structure at the proximal end, beyond spermathecal dilation.

#### Bionomics.

Specimens of *A.bambusana* were collected mainly by light trap, a few were also collected from its host plant bamboo, *Bambusavulgaris* Schrad. ex. J.C. Wendl (Distant, 1918). A cluster of eggs were collected on outer surface of young sprout of bamboo. The eggs were pearl-white in colour and were laid in groups of five or six (Fig. 6).

#### Distribution.

This species is presently only known from India. Distant (1918) described this species from Karnataka. It is also recorded from Tamil Nadu (this paper). Salini and Viraktamath (2015) reported this species from southern part of India with illustration of habitus (dorsal and ventral) and also the external scent efferent system.

### ﻿ Acesines breviceps

Taxon classificationAnimaliaHemipteraPentatomidae

Stål, 1876

3C9A16AA-B6F9-5071-AC9C-960CC73CE0D1

Figs 26–29

Acesines breviceps Stål, 1876: 94–95 (original description). Holotype: ♀, India Orientalis, deposited at the Swedish Museum of Natural History (SMNH).
Acesines breviceps : Atkinson (1888): 132 (redescription); Lethierry and Severin (1893): 171 (catalogue); Distant (1902): 231, fig. 144 (redescription; figures of habitat in dorsal view, and mesothorax, metathorax and abdomen in ventral view; distribution); Kirkaldy (1909): 127 (catalogue); Hasegawa (1962): 8–9, pl.1, fig. 3 (record, specimen label details).

#### Note.

This species is likely to be conspecific with *A.bambusana* because of the similarity in external colouration and structural morphology. The differences (punctures on scutellum, colouration of outer margin of abdominal venter, small spinous projection at the caudal margin of laterotergite VIII) observed upon comparison of images of the female holotype specimen of *A.breviceps* (the photographs were provided by courtesy of Gunvi Lindberg) with female specimens of *A.bambusana*, were found to be insignificant. However, the synonymy of these species cannot be confirmed without the examination of male genitalia structures of both species. Unfortunately, there are no male specimens available for study in the case of *A.breviceps*.

### ﻿ Acesines sordida

Taxon classificationAnimaliaHemipteraPentatomidae

(Kirby, 1891) comb. nov.

0472B562-490E-5F24-82DD-8E25322AAEE3

Raphigaster sordida Kirby (1891): 86–87 (original description). Syntypes: Ceylon (at present Sri Lanka), Pundaloya, deposited at NHM, UK.
Araducta sordida : Distant (1900a): 427 (distribution).
Dunnius sordidus : Distant (1902): 232 (redescription, distribution); Bergroth (1908): 173 (catalogue); Kirkaldy (1909): 137 (catalogue); Chatterjee (1934): 22 (record, host); Chandra (1953): 92 (distribution).

#### Note.

This species was recorded from Aiyur (Tamil Nadu) by Chatterjee (1934) from Sandalwood (*Santalumalbum* L., Santalaceae) and was its first record outside the type locality though this record needs further verification. Since then, no record of this species has been reported in the country. There are only two female syntypes available for this species, deposited at NHM, UK (Figs 30–35). Based on the type images (the photographs were provided by courtesy of M Webb), it is evident that the hollowed out or grooved (or notched) posterior apex of metasternal carina (Fig. 35) (see differential diagnosis under *Acesines* and *Dunnius*) necessitates the transfer of this species from *Dunnius* to *Acesines*. Therefore, *Acesinessordida* comb. nov. is proposed in this paper.

**Figures 26–29.**
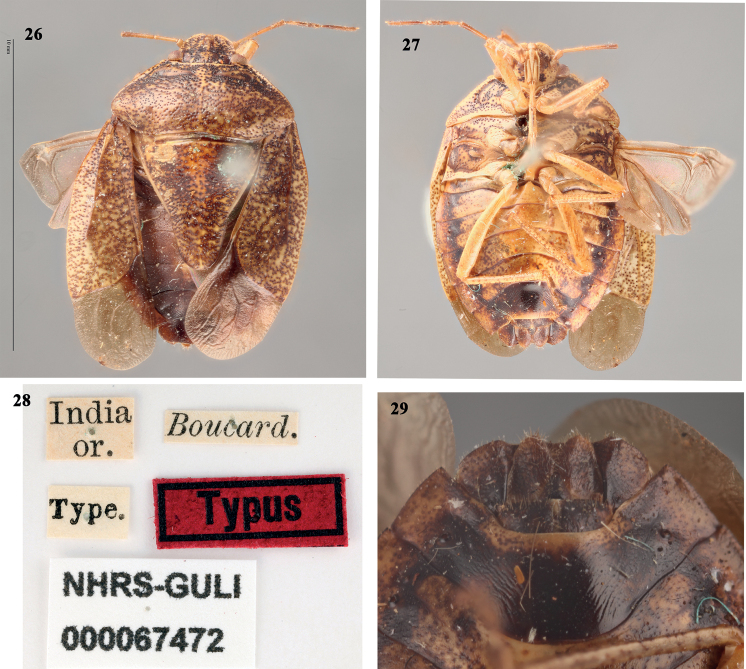
*Acesinesbreviceps* Stål **26** habitus (dorsal) **27** habitus (ventral) **28** label data of *A.breviceps* typus **29** close up of posterior end of ventral side of female abdomen (terminalia). Photographed by Gunvi Lindberg (2021 Naturhistoriska riksmuseet). Original photograph cropped. Made available by the Swedish Museum of Natural History under Creative Commons Attribution 4.0 International Public License, CC-BY 4.0 (https://creativecommons.org/licenses/by/4.0/legalcode).

### ﻿ Dunnius

Taxon classificationAnimaliaHemipteraPentatomidae

Distant, 1902

F497A893-332B-532B-9FB6-A3AE061EC717

Dunnius Distant, 1902: 225 (key to genera), 231–232 (original description). Type species: Raphigastorfulvescens Dallas, 1851: 283, by original designation.
Dunnius : Bergroth (1908): 173 (catalogue); Kirkaldy (1909): 137, 371 (catalogue); Distant (1918): 145 (distribution); Hsiao and Zheng (1977): 99, 103 (key); Rider (2006): 317 (catalogue); Salini and Viraktamath (2015): 12 (key); Rider et al. (2018): 89, 106 (comments on tribal placement).
non Mycterizon Breddin, 1909: 279 (original description). Type species: Araductabella Distant, 1900a: 427, by monotypy. Synonymised by Distant (1918): 145. [removed from synonymy in this paper].

#### Redescription.

***Colouration*.** Body ochraceous, sometimes with two yellow spots dorsally especially on pronotum; membrane pale brown, translucent; legs and antennae pale; abdominal spiracles outlined in black, sometimes with anterior and posterolateral angles of abdominal sternites III–VII black.

***Integument and vestiture*.** Body on dorsal side with brown to black coarse punctures confluent to form narrow black streaks. Ventral side with punctures less dense on thoracic sternites and finer on abdominal sternites; legs with sparse, fine, black punctures.

Body glabrous, antennomeres I–IV with short, semi-erect, golden setae. Labium with sparse, fine, short setae, femora with fine, golden setae, tibiae, and tarsi with moderately dense, short, pale brown semi erect setae. Ventral side of genital capsule with brown, sparse, fine punctures; terminalia with valvifers VIII, laterotergite VIII and laterotergite IX with fine brown punctures. Genital capsule (median region of ventral rim, posterolateral lobes) with short golden setae. Female genitalia (median region of valvifers VIII, valvifers IX laterally, laterotergite VIII and IX caudally) with short, brown, semi-erect setae.

***Structure*.** Body moderate size (8–13 mm long), widely oval, widest across second abdominal segment.

***Head*** above flat, much shorter than width across compound eyes, lateral margins narrowly reflexed, slightly concave in front of compound eyes. Mandibular plates much wider, but as long as clypeus, not meeting in front of clypeus. Antennae with five antennomeres, slender; antennomere I cylindrical, shortest, stoutest, remaining antennomeres cylindrical and slender. Labium reaching or surpassing mesocoxae.

***Pronotum*.** Anterior margin not collar-like, rather thin, median concavity to accommodate posterior margin of head, shallow; anterolateral angles with minute laterally directed teeth. Anterolateral margin obliquely straight, narrowly reflexed; posterior margin nearly straight, posterolateral angles rounded; humeri rounded. Disc of pronotum strongly convex with anterior 2/3 sloping downwards.

***Scutellum*.** Triangular, longer than broad at base; scutellar disc with basal 1/2 gibbous; posteriorly flat. Apex of scutellum narrowly rounded.

***Hemelytra*.** Corium with anterodistal angles rounded, extending well beyond apex of scutellum. Membrane translucent with five or six simple veins, without reticulate venation.

***Thoracic pleuron and sternum*.** Pro- and meso-sternum with median longitudinal carina uniformly narrow, not extending beyond procoxae; metasternal carina developed into a cruciform structure with posterior apex not hollowed out or grooved and not in contact with the basal abdominal tubercle. External scent efferent system (Fig. 57) with peritreme well developed into spout-like extension, reaching 1/3 of metapleural width.

**Figures 30–32.**
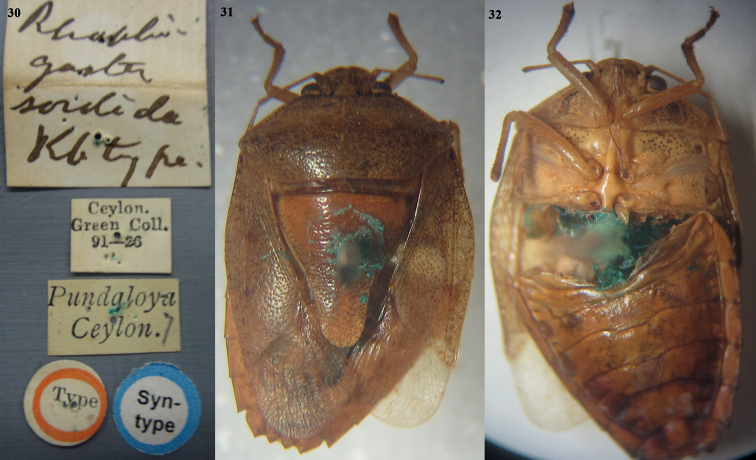
*Acesinessordida* (Kirby, 1891), comb. nov. **30** label data of female syntype 1 **31** female syntype 1 (dorsal habitus) **32** female syntype 2 (ventral habitus). Photographed by Mick Webb, NHM, UK.

***Legs*.** All femora unarmed, cylindrical, rounded in cross-section. All tibiae triangular in cross-section, their outer surfaces sulcate.

***Pregenital abdomen*.** Connexivum slightly exposed. Posterolateral angles of abdominal ventrites angulate. Abdominal venter slightly convex medially, neither grooved nor keeled; ventrite III medially with a prominent tubercle (Fig. 56), apex not in contact with posterior apex of metasternal carina.

***Male genitalia*.** Genital capsule nearly quadrangular with posterolateral lobes (= caudal lobes) well developed, pointed and angular. Dorsal rim sublaterally with convex lobe-like outgrowth nearly concealing the 1+1 triangular tooth-like structure on infoldings of dorsal rim. Ventral rim medially with shallow, roughly rectangular notch. Paramere with crown C-shaped in lateral view, short neck and small plate-like apodeme. ***Phallus*.** Phallotheca broader than long, dorsal region bulbous and ventral margin nearly straight (in lateral view); dorsal conjunctival processes large and membranous subdivided into two processes. Processes of aedeagus fused to form a hollow structure, opening ventrally with broad, oval aperture enclosing short aedeagus; aedeagus tube-like with apex curved.

***Female genitalia*.** Valvifers VIII transverse, broad and roughly quadrangular; valvifers IX single, roughly quadrangular sclerite; laterotergites IX oblique, elongate; short, denticle at caudal apex of laterotergites VIII and laterotergites IX. Spermathecal dilation long, regularly, obliquely fluted (Fig. 51), apical receptacle elongated (Fig. 52), constricted at middle; apical bulbous region with two ductules.

**Figures 33–35.**
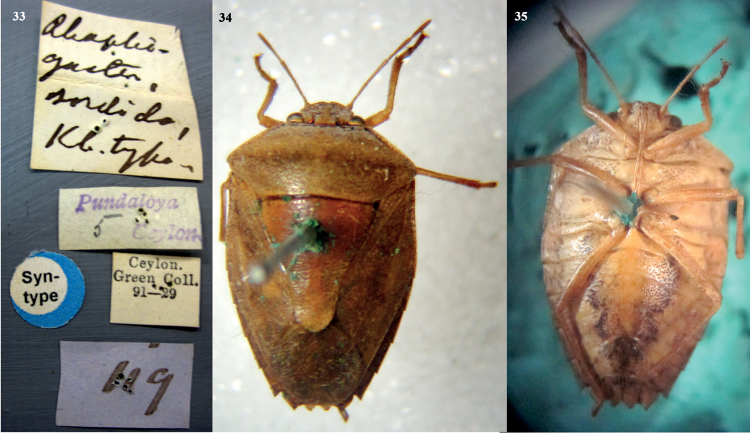
*Acesinessordida* (Kirby, 1891), comb. nov. **33** label data of female syntype 2 **34** female syntype 2 (dorsal habitus) **35** female syntype 2 (ventral habitus). Photographed by Mick Webb, NHM, UK.

#### Differential diagnosis and remarks.

Based on the materials examined under *Dunnius* and *Acesines*, it is found that the species of *Dunnius* closely resembles those of *Acesines*, but the former possesses the cruciform metasternal carina, the posterior end of which is not notched or grooved, which differentiate it from species of *Acesines*. Based on the similarity of male genitalia characters (shape of paramere and shape of caudal lobes of genital capsule), *A.laticeps*, *A.tridentatus*, and *A.trifasciatus* are transferred here from *Acesines* to *Dunnius* (see the differential diagnosis and remarks under *Acesines*). However, the cruciform metasternal carina and the presence or absence of groove or notch at the posterior apex of metasternal carina, could not be verified as neither the original description of those species mentioned it nor were the types examined here. However, the new species of *Dunnius* dealt in this paper (see description *D.barpetensis* sp. nov.) closely resembles *A.laticeps*, *A.tridentatus*, and *A.trifasciatus* in external morphology and male genitalia (characteristic shape of paramere, phallus and caudal lobes of genital capsule) and possesses the cruciform process without the posterior notch or groove reveals that these species are congeneric. This taxonomic decision is also in conformity with studies by Fan (2011), where she tentatively transferred *A.laticeps*, *A.tridentatus*, and *A.trifasciatus* to *Dunnius* in her unpublished thesis and thereby excluded *Acesines* from the Chinese fauna. Another taxonomic decision undertaken in this paper is the transfer of *D.bellus* from *Dunnius* to *Mycterizon*. Investigation based on external morphology and genitalia (see under the redescription of *Mycterizon*) revealed that it is not congeneric and therefore removed from this genus and *Mycterizonbellus* (Breddin, 1909) is proposed after the status of *Mycterizon*Breddin (1909) is reinstated. Hence *Dunnius* is presently proposed to contain six species: *D.fulvescens*, *D.minor*, *D.laticeps*, *D.tridentatus*, *D.trifasciatus*, and *D.barpetensis*.

#### Etymology.

Neither the etymology nor the gender of the name *Dunnius* were explicitly given in the original description. The name *Dunnius* is a Latin word, which is masculine according to the Article 30.1 of ICZN (1999).

### ﻿ Dunnius barpetensis

Taxon classificationAnimaliaHemipteraPentatomidae

Salini & Rabbani sp. nov.

4CB1AA5A-E80B-5B22-844E-4079407A6B9B

https://zoobank.org/75CAC1BD-8E00-4BD0-8461-49A76BA17779

Figs 36–41 , 42–48 , 49–52

#### Type locality.

India, Assam, Barpeta, Sorbhog, Ramie Research Centre, 26°31'25.3"N, 90°53'09.7"E.

#### Type material.

***Holotype***: ♂ (NIM), ‘INDIA: Assam/ Barpeta, Sorbhog/ Ramie Research Centre/26°31'25.3"N, 90°53'09.7"E/ 09.iii.2021, Rabbani, M. K.// HOLOTYPUS/ *DUNNIUS BARPETENSIS* sp. nov./det. Salini. S., 2022 [p, red label]’. Holotype is pinned, the dissected male genitalia placed in a plastic microvial with glycerol attached to the same pin, the detached right forewing glued on card. ***Paratype*** (1♀): **India**: Meghalaya/ Anggalanggri, South Garo hills/ 25°20'53.0"N, 90°13'37.8"E/ 20.iii.2021, Rabbani, M. K.(NIM), dissected female genitalia placed in a glass microvial with glycerol attached to the same pin; abdominal tergites and ventrites glued on card; segment V of right antenna, whole tarsi including apical claws of right foreleg and left hind leg lost. The paratype bears the following identification label: ‘PARATYPUS / *DUNNIUS BARPETENSIS* sp. nov. / det. Salini. S., 2022 [p, yellow label]’.

#### Description.

***Colouration*.** Body above pale brown to ochraceous; head including antennae, 1/2 of anterolateral margins of pronotum and dorsum of scutellum pale white. Ocelli reddish. 1+1 small, round pale white spots located on posterior margin of pronotal calli. Membrane pale brown, translucent. Ventral side including legs creamy white except the following: apex of labiomere IV, apical 1/2 of tarsal claws, spiracular outline, minute spot at the antero- and postero-lateral angles of abdominal sternites III–VII, black.

***Integument and vestiture*.** Body above covered with coarse, black punctures, densely concentrated towards anterior margin of pronotum and lateral margins of scutellum. Ventral side of body with black punctures mostly coarse and dense on thoracic sternites. Legs with minute black punctures sparsely distributed. The punctures finer on abdominal sternites, appear as four faint, indistinct streaks, gradually disappearing towards posterior 1/2. Ventral side of genital capsule with brown, sparse, fine punctures; terminalia with valvifers VIII, laterotergite VIII and laterotergite IX with fine, brown punctures.

Body glabrous, except the following: antennae and femora with fine, semi-erect, short setae; labium with sparse, golden setae; tibiae with dense, brown, short, semi erect setae on ventral side and dorsally with moderately elongate brown setae, dense mat of setae at the posterior end of tibiae; tarsomeres on dorsal side with moderately elongate golden setae and dense mat of short, golden setae on ventral side of all tarsomeres. Genital capsule (median region of ventral rim, posterolateral lobes and the infoldings of ventral rim with cushion-like region on either side of the median notch) with short, golden setae. Female genitalia (median region of valvifers VIII, valvifers IX laterally, laterotergite VIII and IX caudally) with short, brown semi-erect setae.

**Figures 36–41.**
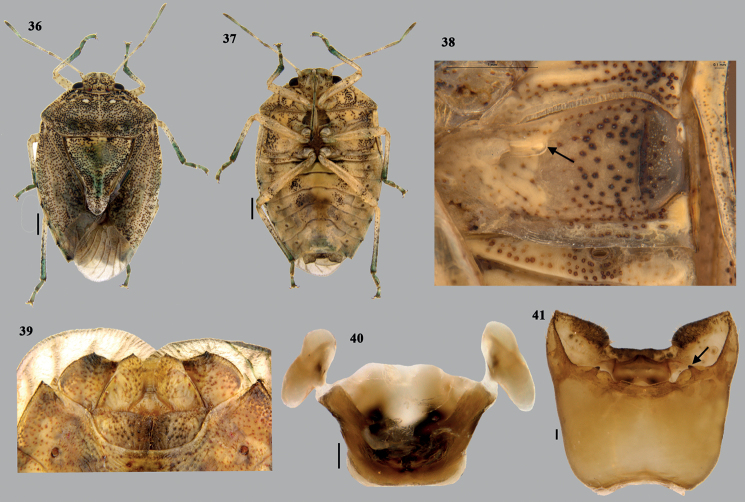
*Dunniusbarpetensis* Salini & Rabbani, sp. nov. **36** habitus (dorsal) **37** habitus (ventral) **38** external scent efferent system (arrow showing the peritreme) **39** close up of posterior end of ventral side of female abdomen (terminalia) **40** articulatory apparatus **41** genital capsule (dorsal) (arrow shows the triangular tooth-like structure). Scale bars: 1 mm (**36, 37**); 0.1 mm (**38–41**).

***Structure*.** Head above flat, sloping downwards, much shorter than wide, lateral margins narrowly reflexed, slightly concave in front of compound eyes. Mandibular plates more than twice as wide as clypeus, but nearly as long as clypeus, not meeting in front of clypeus. Clypeus narrow throughout its length. Compound eyes moderately large, rounded, protruding out of the head outline in most of their width. Ocelli small, situated posteriorly near to posterior margin of head. Antenniferous tubercles short. Antennae with five antennomeres, slender, antennomeres from shortest to longest: I<II_b_<II_a_<IV<III; antennomere I approximately reaching apex of head, shortest and stoutest, antennomere IIa to cylindrical and slender and antennomere IV slightly thickened towards apex. Bucculae short, low, anterior apex angulate and sometimes developed into minute tooth. Labium passing mesocoxae, labiomere I as long as bucculae.

***Pronotum*.** Anterior margin thin, with median concavity to accommodate posterior margin of head, shallow; anterolateral angles with tooth minute or indistinct laterally directed. Anterolateral margin obliquely straight, narrowly reflexed; posterolateral margin narrowly concave; posterior margin nearly straight, posterolateral angles rounded; humeri rounded. Disc of pronotum convex with anterior 2/3 sloping downwards.

**Figures 42–48.**
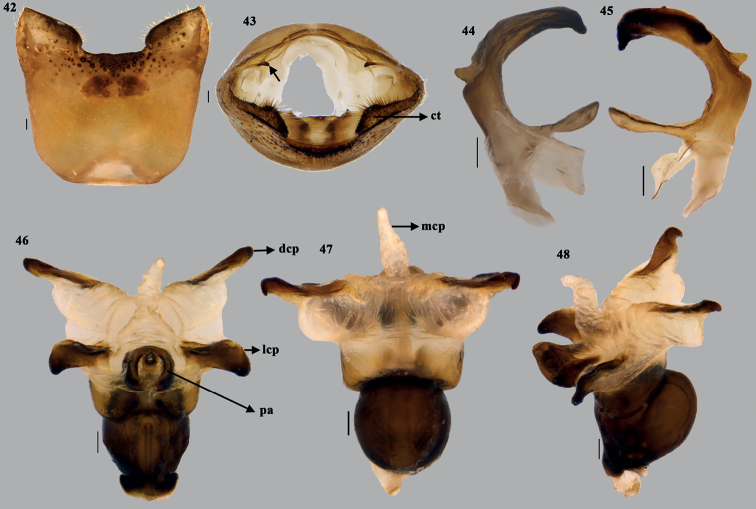
*Dunniusbarpetensis* Salini & Rabbani, sp. nov. **42** genital capsule (ventral) **43** genital capsule (caudal) **44, 45** parameres (different planes) **46** phallus (ventral) **47** phallus (dorsal) **48** phallus lateral. Scale bar: 0.1 mm. Abbreviations: ct–cushion-like triangulate region; pa–processes of aedeagus; dcp–dorsal conjunctival processes; lcp–lateral conjunctival processes; mcp–median conjunctival processes.

***Scutellum*.** Subtriangular, longer than broad at base; basal 1/2 of scutellar disc, gibbous; posterior 1/2, flat; abruptly narrowed at 1/3 from apex; caudal apex of scutellum narrowly rounded.

***Hemelytra*.** Corium with anterodistal angles angulate, extending well beyond apex of scutellum. Membrane translucent with six or seven simple veins, without reticulate venation.

***Thoracic pleuron and sternum*.** Pro- and meso-sternum with median longitudinal carina narrow throughout the length except slightly high at anterior apex, not extending beyond procoxae; posteriorly in contact with cruciform metasternal carina (Fig. 56); posterior apex of metasternal carina not hollowed out or grooved and not in contact with the apex of basal abdominal tubercle. External scent efferent system with peritreme well developed into spout-like, nearly reaching 1/3 of metapleural width.

***Legs*.** Outer surface of tibiae sulcate. All tarsi with tarsomere II shortest, dorsally regularly rounded, tarsomeres I and III subequal.

***Pregenital abdomen*.** Connexivum scarcely exposed. Posterolateral angles of abdominal ventrites with tooth minute or indistinct. Abdominal venter slightly convex medially, neither grooved nor keeled; ventrite III (= second visible abdominal segment) medially with tubercle short, apically rounded and stout.

**Figures 49–52.**
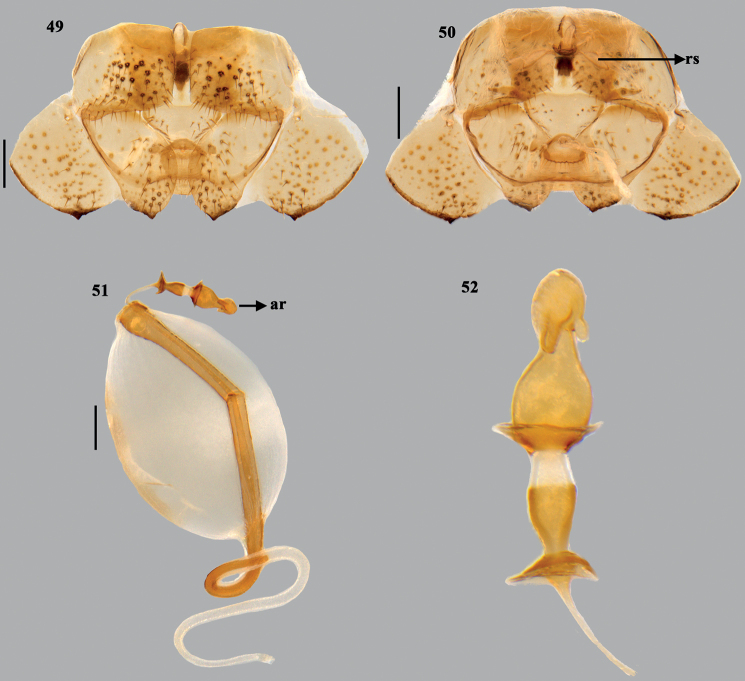
*Dunniusbarpetensis* Salini & Rabbani, sp. nov. **49** terminalia (dorsal) **50** terminalia (ventral) **51** spermatheca **52** spermathecal pump. Scale bars: 0.5 mm (**49–51**). Abbreviations: ar–apical receptacle; rs–ring sclerites.

***Male genitalia*** (Figs 40–48). Genital capsule nearly quadrangular with posterolateral lobes (= caudal lobes) well developed and angular; apex of posterolateral lobes somewhat pointed. Dorsal rim widely excavated with narrow median emargination, sublaterally with convex lobe-like outgrowth nearly concealing the 1+1 triangular tooth-like structure (Fig. 41) on infoldings of dorsal rim. Dorsal sinus of posterior aperture semi-circular; infoldings of dorsal rim with 1+1 triangular, sclerotised, short, tooth-like projection on either side of the dorsal sinus. Ventral rim medially with shallow, roughly rectangular notch; infoldings of ventral rim well developed, either side of median notch developed into thick cushion-like triangulate (ct) region, possessing short, dense, semi-erect setae. Paramere with crown C-shaped in lateral view, short neck and small plate-like apodeme. ***Articulatory apparatus*.** Support bridge complex excavated cup-like, cone-like capitate processes attached with short dorsal connectives. ***Phallus*.** Phallotheca broader than long, dorsal region bulbous (Fig. 48) and ventral margin nearly straight (in lateral view); apical part of phallotheca broader than basal part, pair of short, stout, sclerotised, ventrolateral tubercles at the posterior end of phallotheca; conjunctival processes large, membranous subdivided into a pair of large, partly membranous, dorsal conjunctival processes (dcp), one median, short, membranous process (mcp) and pair of partly membranous, lateral conjunctival processes (lcp). Apex of dorsal conjunctival process smooth, curved into hook-like and that of lateral conjunctival processes rounded with minute serrations. Processes of aedeagus (pa) fused to form hollow structure, opening ventrally with broad oval aperture and enclosing short aedeagus; aedeagus tube-like with apex curved and phallotreme rounded.

***Female genitalia*.** (Figs 39, 49–52). Valvifers VIII transverse, broad and roughly quadrangular, with medial margins nearly straight; inner posterolateral angles angulate; posterior margin straight; valvifers IX single, nearly quadrangular sclerite; laterotergites IX oblique, elongate with inner margin broadly arcuate and outer margin more or less straight, caudal margin moderately sclerotised, caudal apex with short denticle; laterotergite VIII subquadrate, caudal margin slightly convex, with short sclerotised denticle on caudolaterally. A pair of ring sclerites (rs) elongate lanceolate. ***Spermatheca*.** Spermathecal dilation long, regularly and obliquely fluted; distal spermathecal duct narrow and shorter than proximal one; proximal spermathecal duct moderately broad and tubular; proximal flange shorter than distal flange; apical receptacle (ar) elongate, constricted at middle, large, bulbous base connected to short distal apical bulbous region by short, narrow region; apical bulbous region with two ductules, of which one elongate and other short, both facing towards distal rim and not reaching distal rim.

#### Measurements

**(mm).** Males (*n* = 1); Body length 12.77; head: length 1.84, width (including eyes) 3.01, interocular width 1.63; lengths of antennomeres: I – 0.50, II – 1.25, III – 1.05, IV – 1.67, V – 1.42; lengths of labiomeres: I– 0.89, II– 1.39, III– 0.83, IV– 0.74; pronotum: length 2.98, width (including humeri) 6.74; scutellum: length 4.63, width (at basal angles) 4.45.

Females (*n* = 1); Body length 14.12; head: length 1.92, width (including eyes) 3.24, interocular width 1.88; lengths of antennomeres: I – 0.57, II – 1.35, III – 1.16, IV – 1.62, V – 1.43; length of labiomeres: I – 1.02, II – 1.44, III – 1.14, IV – 0.71; pronotum: length 3.45, width (including humeri) 7.15; scutellum: length 5.17, width (at basal angles) 4.63.

#### Differential diagnosis.

This species resembles *D.laticeps*, *D.tridentatus*, and *D.trifasciatus* in external morphology and colouration and therefore difficult to differentiate unless the male genitalia has been examined. The characteristic shape of genital capsule (dorsal rim, ventral rim, 1+1 denticle-like structure on infoldings of dorsal rim), shapes of paramere and phallus can differentiate this species from its congeners.

#### Etymology.

The specific epithet, *barpetensis*, is based on the name of the type locality; adjective.

#### Bionomics.

The species was collected on bamboo, *Bambusavulgaris* Schrad. & J.C. Wendl.

#### Distribution.

This species is at present known only from Assam and Meghalaya.

### ﻿ Dunnius fulvescens

Taxon classificationAnimaliaHemipteraPentatomidae

(Dallas, 1851)

0C1A3795-3285-5DF4-BF2B-A0E44F3B341A

Figs 53–55 , 56–62

Rhaphigaster (Raphigaster) fulvescens Dallas, 1851: 283 (original description). Holotype: ♀ (NHM: ? lost, M Webb, pers. comm.).
Rhaphigaster fulvescens : Dohrn (1859): 18 (checklist); Walker (1867): 373 (list); Stål (1876): 129 (incertae sedis); Lethierry and Severin (1893): 200 (catalogue).
Plexippus fulvescens : Distant (1900b): 387 (distribution); Distant (1901): 102 (distribution).
Dunnius fulvescens : Distant (1902): 232, fig. 145 (redescription; figures of habitat in dorsal view, mesothorax, metathorax and abdomen in ventral view; distribution); Kirkaldy (1909): 137 (catalogue); Chatterjee (1934): 22 (distribution, record from Assam and Naga hills, host); Chandra (1953): 92 (record from Tamil Nadu); Mathur and Singh (1960): 20 (host); Yang (1962): 117, 124–125 (distribution, key to tribe Antestiarini); Jiang (1985): 67 (distribution in China); Zhang et al. (1987): 144 (key); Zhang and Lin (1990): 3 (distribution in China); Yang (1993): 213 (distribution in China); Zhang et al.(1995): 58, pl. 14, fig. 143 (description); Rider et al. (2002): 139 (Chinese distribution, checklist); Chakraborty and Ghosh (2003): 517 (checklist for Sikkim); Rider (2006): 317 (catalogue).

#### Type locality.

Country unknown (Dallas, 1851).

#### Type material.

**Figures 53–55.**
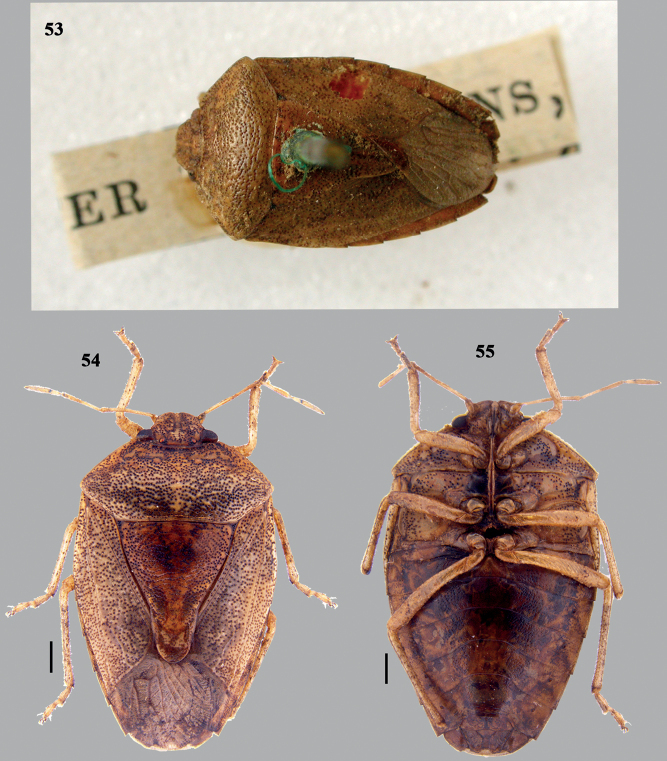
*Dunniusfulvescens* (Dallas) **53** syntype image (dorsal habitus), courtesy: P. Kment **54** habitus (dorsal) **55** habitus (ventral). Scale bars: 1 mm.

Not examined, only image of type was seen. The original description was based on female specimen (or specimens?). The type is supposedly deposited in NHM, London; however, it appears to be lost at present (M Webb, pers. comm.). The image of the type (Fig. 53) was captured prior to its loss, provided by P Kment.

#### Material examined.

**India: Arunachal Pradesh**: 1♀, Pasighat, 2–11 May 2014, Veena Kumari, K., ICAR-NBAIR (NIM).

#### Redescription.

***Colouration*** Body above pale brown to ochraceous. Ocelli reddish. 1+1 small, round, yellow spots on anterior 1/2 of pronotum, located on posterior margin of pronotal calli. Small, pale yellow irregular markings scattered on the pronotal disc. Membrane translucent with brown veins. Antennae and legs paler than dorsal colouration. Ventral side concolourous to dorsal except the following: four narrow broken longitudinal stripes (two laterally and another two sublaterally, formed from coarse, black punctures), spiracular outline, posterolateral angles of abdominal sternites III–VII, moderately large and round spot on mesosternum, apex of labiomere IV, black.

***Integument and vestiture*.** Body above (Fig. 54) covered with coarse, black punctures, dense on head (dorsally), pronotum and scutellum; clusters of punctures sometimes forming irregular markings especially on head and pronotum; punctures on hemelytra are less dense; head (ventrally), thoracic pleura and abdominal sternites (four broken longitudinal stripes) with coarse, brown punctures; connexivum with fine, brown punctures.

Body glabrous except antennae and legs moderately pilose. Terminalia of female genitalia with valvifers VIII and IX, laterotergites VIII and IX provided with sparse, coarse, brown punctures.

***Structure*.** See the generic redescription.

***Male genitalia*.** Unknown.

***Female genitalia*** (Figs 58–62). Valvifers VIII transverse, broad and quadrangular, with medial margins straight; inner posterolateral angles angulate; posterior margin nearly straight; valvifers IX single, transverse, large subtrapezoidal sclerite with caudal margin concave; laterotergites IX oblique, elongate; outer margins and caudal 1/2 of inner margin straight; caudal margin of laterotergites IX irregularly truncated; laterotergites VIII subquadrangular with outer margins more or less obliquely straight; caudo-lateral margin with indistinct, denticle, black. A pair of ring sclerites (rs) (Fig. 60) elongate, roughly oblong. ***Spermatheca*** (Fig. 61). Spermathecal dilation long, regularly, fluted; distal spermathecal duct narrow, shorter than proximal one; proximal spermathecal duct thick, elongate and tubular, sclerotised rod at the middle of lumen of spermathecal dilation twisted at proximal end beyond the dilation; proximal flange nearly 1/2 of distal flange; apical receptacle drop-shaped constricted towards apex, bearing three ductules apically, facing towards distal rim and not reaching distal rim.

#### Measurements

**(mm).** Females (*n* = 1); Body length 11.16; head: length 1.57, width (including eyes) 2.72, interocular width 1.53; lengths of antennal segments: I –0.43, II – 1.25, III – 1.03, IV – 1.46, V– 1.31; length of labial segments: I –0.60, II – 1.25, III – 0.87, IV – 0.64; pronotum: length 2.66, width (including humeri) 6.47; scutellum: length 4.77, width (at basal angles) 4.04.

#### Differential diagnosis.

This species resembles other members of this genus externally but the genitalia characters like the laterotergites IX having the caudal margin irregularly truncated, smooth, without any denticle and the drop-shaped apical receptacle differentiates this species from its congeners.

#### Distribution.

India: Sikkim (Mungphu), Tamil Nadu (Udhagamandalam = Ootacamund) (Distant, 1900b), Arunachal Pradesh (Pasighat) (new record); Burma (Distant, 1902); China (Rider, unpublished catalogue).

**Figures 56–62.**
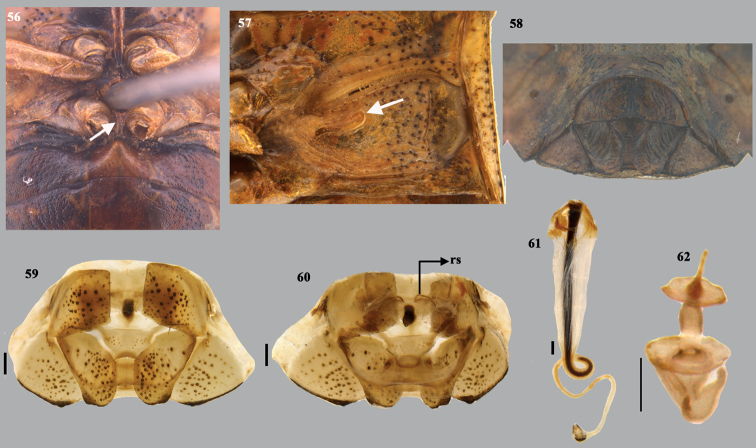
*Dunniusfulvescens* (Dallas) **56** metasternal carina with cruciform posterior apex (arrow showing the cruciform posterior apex) and basal abdominal tubercle **57** external scent efferent system (arrow showing the peritreme) **58** close up of posterior end of ventral side of female abdomen (terminalia) **59** terminalia (dorsal) **60** terminalia (ventral) **61** spermatheca **62** spermathecal pump. Scale bar: 0.25 mm. Abbreviation: rs–ring sclerites.

### ﻿ Dunnius laticeps

Taxon classificationAnimaliaHemipteraPentatomidae

(Zheng & Liu, 1987) comb. nov.

D8355E55-3837-54DD-B09F-4699388C9D3B

Acesines laticeps Zheng & Liu, 1987: 187–188, 191, figs 25–29 (original description). Holotype: ♂, Yunnan (Mengla), China (Nankai University), 17.IX.1979, Zou Huan-guang leg.
Acesines laticeps : Zhang and Lin (1990): 2 (distribution); Rider et al. (2002): 135 (Chinese distribution); Rider (2006): 259 (catalogue).

#### Type material.

Not examined. The description was based on a single male specimen (holotype) from Yunnan, China and is deposited in Nankai University as per the original description.

#### Note.

This species was originally described in the genus *Acesines* Stål, but the illustration of dorsal habitus and the male genitalia which were provided in the original description by Zheng and Liu (1987) indicate that it is not congeneric with members of *Acesines*, but rather closely resembles members of *Dunnius*, especially *D.barpetensis* sp. nov. described in this paper. Fan (2011) transferred *A.laticeps* with the other two species, *A.tridentatus* and *A.trifasciatus*, from *Acesines* to *Dunnius* based on length of labium and mesosternal ridge thickness, and redescribed and illustrated the male and female genitalia of *D.laticeps*. Another important diagnostic for differentiating the members of *Acesines* from *Dunnius* is the notch at the posterior apex of metasternum, which is designed to accommodate the apex of short basal abdominal tubercle, and this is evident in the holotype image of the type species, *A.breviceps* (the photographs were provided by courtesy of Gunvi Lindberg). This was mentioned by Distant (1902) as a genus diagnostic for *Acesines* as “… abdomen with a distinct basal tubercle or spine which touches the metasternum ...”, but in the case of *Dunnius*, the metasternum forming a central cruciform process and not notched posteriorly and the basal abdominal tubercle rather short, approximately reaching metasternal process (indicates that it is not in contact with metasternum). Although the original description of *D.laticeps* did not mention these diagnostics, the ventral habitus image provided by Fan (2011: 573, fig. 215) indicates that the apex of basal abdominal tubercle of *D.laticeps* neither accommodated by or nor in contact with metasternum. Moreover, the similarity of male genitalia like the shape of paramere, genital capsule (shape of caudolateral lobes, ventral and dorsal rim) and shape of phallus with *Dunniusbarpetensis* sp. nov. Based on the above evidence, this species is transferred from *Acesines* to *Dunnius* in this work.

### ﻿ Dunnius tridentatus

Taxon classificationAnimaliaHemipteraPentatomidae

(Xiong & Liu, 1995) comb. nov.

FE6DE32E-C313-5D0B-AD07-6472D8168103

Acesines tridentatus Xiong & Liu, 1995: 264–266, fig. 2a–d (original description). Holotype: ♂, Yunnan (Tengchong), China (Kunming Institute of Zoology, the Chinese Academy of Science). Paratype: ♀, Yunnan (Tengchong), 27.IX.1981, Wang Jixian leg.
Acesines tridentatus : Rider et al. (2002): 135 (Chinese distribution); Rider (2006): 259 (catalogue); Li et al. (2015): 267 (distribution in China).

#### Type material.

Not examined. The description was based on one male and female each from Tengchong, China and is deposited in Kunming Institute of Zoology, the Chinese Academy of Science as per the original description.

#### Note.

See the note under *D.laticeps* comb. nov. This species was originally described in the genus *Acesines* Stål, but similar to members of *Dunnius* in external morphology and male genitalia like the shape of paramere, genital capsule (shape of caudolateral lobes, ventral and dorsal rim) and shape of phallus similar to that of *D.barpetensis* sp. nov. The absence of metasternal notch and the short basal abdominal tubercle, which is not in contact with metasternum (see under the note of *D.laticeps*), which is evident from the description and ventral habitus image of this species by Fan (2011: 573, fig. 219). This also indicates that *Acesinestridentatus* is congeneric with *Dunnius* and hence transferred from *Acesines* to *Dunnius* in this paper.

### ﻿ Dunnius trifasciatus

Taxon classificationAnimaliaHemipteraPentatomidae

(Xiong & Liu, 1995) comb. nov.

5A5C7408-BC27-565F-9882-C8318AB37335

Acesines trifasciatus Xiong & Liu, 1995: 263–264, fig. 1a–d (original description). Holotype: ♂, Yunnan (Mengzhe), China, 19.VI.1984, Xiong Jiang leg. (Kunming Institute of Zoology, Chinese Academy of Science).
Acesines trifaciatus Xiong & Liu, 1995: 266 (lapsus calami).
Acesines trifaciatus : Rider et al. (2002): 135 (Chinese distribution); Rider (2006): 259 (catalogue); Li et al. (2015): 267 (distribution in China).

#### Type material.

Not examined. The description was based on a single male specimen (holotype) Yunnan (Mengzhe), China and is deposited in Kunming Institute of Zoology, the Chinese Academy of Science as per the original description.

#### Note.

See the note under *D.laticeps* comb. nov. This species was originally described in *Acesines* Stål, but is similar to members of *Dunnius* in external morphology and male genitalia in the shapes of paramere, genital capsule (shape of caudolateral lobes, ventral and dorsal rims), and shape of phallus, which are similar to that of *D.barpetensis* sp. nov. The absence of metasternal notch and the short basal abdominal tubercle, which is not in contact with metasternum (see under the note of *D.laticeps*), is evident from the description and ventral habitus image of this species by Fan (2011: 575, fig. 221). This also indicates that *Acesinestrifasciatus* is congeneric with *Dunnius* and hence transferred from *Acesines* to *Dunnius* in this paper.

### ﻿ Mycterizon

Taxon classificationAnimaliaHemipteraPentatomidae

Breddin, 1909 stat. rev.

E75FFDE6-5DD2-577A-AD92-8283139AC1FF

Mycterizon Breddin, 1909: 279 (original description). Type species: Araductabella Distant, 1900a: 427, by monotypy. Synonymized with Dunnius by Distant (1918): 145.

#### Redescription.

***Colouration*.** Body ochraceous, with irregular black markings on pronotum, scutellum and hemelytra; membrane smoky brown, translucent; legs and antennae pale white; apex of labium, small, transverse line below the spiracles on either side of abdomen, small, irregular spots laterad to spiracle on either side of abdomen, anterior and posterior lateral angles of abdominal sternites III–VII, black.

***Integument and vestiture*.** Body on dorsal side with black, coarse punctures confluent to form narrow black transverse streaks especially on pronotum, sometimes forming medium to large, irregular spots; fine punctures on connexivum. Ventral side with punctures more concentrated laterad; punctures fine and less dense medially; femora with dense, coarse punctures and tibiae with fine, black punctures. Genital capsule (ventrally) including posterolateral lobes with fine pale brown punctures; valvifers VIII and laterotergite VIII with sparse, coarse, brown punctures.

Dorsum glabrous, antennomeres I–IV and labium with short, semi-erect, dense golden setae; legs with moderately elongate golden setae; ventral side of abdomen with dense short, golden pubescence. Genital capsule (dorsal rim, posterolateral lobes and the caudal 1/2 of genital capsule on ventral side) with moderately elongate, dense, semi erect, golden setae. Female genitalia (valvifers VIII, valvifers IX, laterotergite VIII and IX) with moderately long, golden semi-erect setae.

***Structure*. *Head*** above flat, as broad as 1.5 times the head length, lateral margins not reflexed, distinctly concave in front of compound eyes, outline of anterior part of head disc inverted U-shaped, beyond the concavity on lateral margins, in front of compound eyes; dorsum of head disc with transverse impression medially along the imaginary transverse line connecting the anterior margins of compound eyes and another 1+1 short, C-shaped impression adjacent to inner margins of compound eyes. Head length behind compound eyes much shorter and accommodated in the shallow median concavity of anterior margins of pronotum. Mandibular plates nearly twice as wide as width of clypeus, moderately narrowed towards apex, nearly as long as clypeus, not meeting in front of clypeus. Compound eyes protruded and stylate-like. Antennae with five antennomeres, slender; antennomere I cylindrical, shortest and stoutest, nearly reaching apex of head, remaining antennomeres cylindrical and slender. Labium short, reaching mesocoxae.

***Pronotum*.** Anterior margin collar-like, slightly concave medially, anterior pronotal margin including the minute, laterally directed denticle, nearly as wide as head width including compound eyes. Anterolateral margin obliquely straight, not reflexed, smooth; posterior margin concave medially, posterolateral margins obliquely straight, posterolateral angles rounded; humeri rounded. Disc of pronotum strongly convex with anterior 2/3 sloping downwards.

***Scutellum*.** Longer than broad at base; scutellar disc slightly gibbous basally; lateral margins slightly convex in frenal portion; posterior 1/2 of scutellum, beyond frena, broad and nearly U-shaped, with apex broadly rounded.

***Hemelytra*.** Corium with anterodistal angles rounded, extending beyond apex of scutellum. Membrane translucent with seven or eight simple veins, without reticulate venation.

***Thoracic pleuron and sternum*.** Mesosternum with median longitudinal carina uniformly narrow, not extending beyond procoxae; metasternal carina low, less developed. External scent efferent system with peritreme well developed, short, nearly reaching mid-metapleuron, spout-like, obliquely elevated to pleural surface (Fig. 65), the distal end of peritreme slightly raised, forming plate-like structure (unlike adjacent to pleuron), reaching middle of metapleural width.

***Legs*.** All femora unarmed, cylindrical and rounded in cross-section. All tibiae with median longitudinal sulcation, rounded beneath. tarsi dorsally regularly rounded, tarsomere II shortest and tarsomere I and III subequal.

***Pregenital abdomen*.** Body moderate size (8–10.45 mm long), oblong, abdomen nearly as wide as width across pronotal humeri and possesses more or less uniform width throughout. Connexivum usually not exposed. Sternites smooth, devoid of furrows or ridges; basal abdominal sternites lacking tubercle or spine or distinct groove; posterolateral angles of sternites III–VII either angulate or sometimes with short, stout, an blunt tooth.

***Male genitalia*.** Genital capsule nearly quadrangular with posterolateral lobes well developed and broadly rounded. Dorsal rim widely and deeply excavated concave with nearly straight middle margin. Ventral rim medially shallowly excavated and broadly concave. ***Paramere*.** Sclerotised, crown broad, subquadrate. Phallotheca longer than broad, with thecal process slightly developed; four pairs of conjunctival processes; processes of aedeagus fused mid longitudinally, partially enclosing aedeagus.

***Female genitalia*.** Valvifers VIII transverse, broad and roughly quadrangular, with medial margins nearly straight; valvifers IX single, transverse, broad sclerite; laterotergites IX oblique, elongate; laterotergite VIII subtriangular with smooth caudal margin; spermatheca with proximal end of sclerotised rod, at the middle of lumen of spermathecal dilation, curved upwards; apical receptacle orbicular with three elongate ductules.

#### Differential diagnosis and remarks.

Breddin (1909) proposed *Mycterizon* to accommodate *Dunniusbellus* by understanding the fact that this species is unrelated from members of *Dunnius* and the need of a separate genus to accommodate the same. However, Distant (1918) synonymised *Mycterizon* with *Dunnius* without justifying his action. By studying the external morphology, male and female genitalia of the members of *Dunnius*, it was found that *D.bellus* is quite different from members of *Dunnius* in possession of the specific morphological characters, which are as follows: the shape of head (much broad, nearly 1.5 times as wide as long) and scutellum (broad, posteriorly U-shaped apex), short labium (reaching to mesocoxae), external scent efferent system (spout-shaped peritreme with distal end protruding, elevated from the pleural surface; peritreme extends to middle of metapleuron), presence of a narrow, median, longitudinal ridge on mesosternum (not extending anteriorly beyond procoxae), absence of a cruciform metasternal carina (without a notch or groove at the posterior end) apart from the male genitalia characters such as shape of genital capsule including dorsal rim (slightly concave medially, without denticle on infoldings of ventral rim) and ventral rim (broadly concave) and shape of paramere (broad crown possessing stout finger-like straight process). Therefore, the status of *Mycterizon* is reinstated from *Dunnius*Distant (1902) to accommodate *Dunniusbellus* (Distant, 1902) and thereby the status of *Mycterizonbellus* (Distant, 1902) Breddin 1909 is reinstated from *Dunniusbellus* (Distant, 1902).

### ﻿ Mycterizon bellus

Taxon classificationAnimaliaHemipteraPentatomidae

(Distant, 1902) stat. rev.

73F96188-B34E-5CBF-9E5C-B93F54979C9E

Figs 63–68 , 69–75 , 76–79 , 80–85

Araducta bella Distant, 1900a: 427 (original description). Syntype: ♂, Punduloya, Ceylon, Atkinson Coll. 92–6, 116 (NHM), London, UK, lectotype (present designation); Syntype: ♀, Punduloya, Ceylon, Atkinson Coll. 92–6, 116 (NHM), London, UK, paralectotype (present designation).
Dunnius bellus Distant, 1902: 233, fig. 146 (redescription, figures of habitat in dorsal view, and mesothorax, metathorax and abdomen in ventral view; distribution); Bergroth (1908): 173 (catalogue); Kirkaldy (1909): 137 (catalogue); Distant (1918): 145 (synonymisation, additional distribution); Chatterjee (1934): 22 (host record, new records); Chandra (1953) (distribution); Mathur and Singh (1960): 20 (host); Salini and Viraktamath (2015): 12 (key to genera), 24 (checklist), 46, fig. 54 (illustration of dorsal habitus), 68, fig. 185 (illustration of external scent efferent system); Salini (2019): 126, fig. 8.20 (illustration of dorsal habitus).
Mycterizon bellus Breddin (1909): 279 (synonymisation); Kirkaldy (1909): 371 (catalogue).

#### Type locality.

*Araductabella*. Ceylon (= Sri Lanka), Punduloya. *Dunniusbellus*. Ceylon (= Sri Lanka), Punduloya.

**Figures 63–68.**
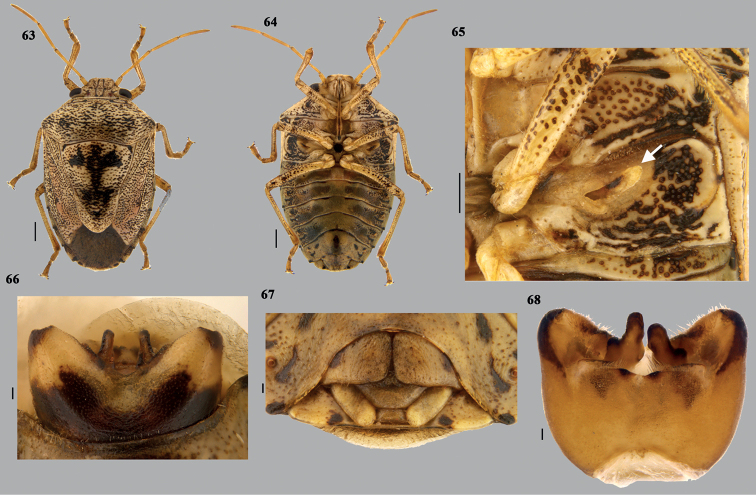
*Mycterizonbellus* (Distant), stat. rev. **63** habitus (dorsal) **64** habitus (ventral) **65** external scent efferent system (arrow showing the peritreme) **66** close up of posterior end of ventral side of male abdomen **67** close up of posterior end of ventral side of female abdomen (terminalia) **68** genital capsule (dorsal). Scale bars: 1 mm (**63, 64**); 0.5 mm (**65**); 0.1 mm (**66–68**).

**Figures 69–75.**
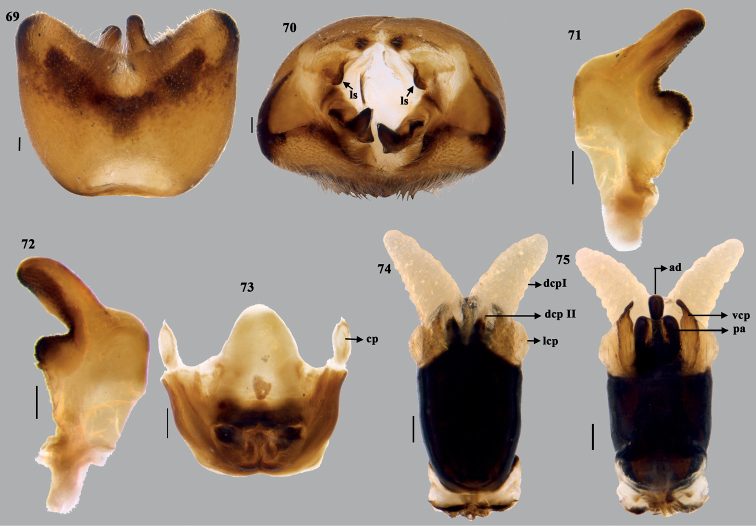
*Mycterizonbellus* (Distant), stat. rev. **69** genital capsule (ventral) **70** genital capsule (caudal) **71, 72** paramere **73** articulatory apparatus **74** phallus (dorsal) **75** phallus (ventral). Scale bar: 0.1 mm. Abbreviations: ad – aedeagus; cp – capitate processes; ls – lobe-like ingrowths; pa – processes of aedeagus; dcp I – dorsal conjunctival processes (inner one); dcp II – dorsal conjunctival processes (outer one); lcp – lateral conjunctival processes; vcp – ventral conjunctival processes.

#### Type material.

Not examined, only type image was seen. The original description of *A.bella* is based on a male specimen whereas that of *D.bellus* was based on a female specimen. The syntype male specimen of *Araductabella* Distant, 1900a: 427 (Ceylon: Punduloya, Atkinson Coll., 92–6, NHMUK 013588914) herein designated as lectotype (Figs 80–82) and the female syntype specimen of *Araductabella* Distant, 1900a: 427 (Ceylon: Punduloya, Atkinson Coll., 92–6, NHMUK 013588915) is herein designated as paraletotype (Figs 83–85). Additionally, 31 specimens of this species are available in NHM (M Webb, pers. comm.). The image of the type is provided by courtesy of M Webb.

#### Material examined.

**India: Andhra Pradesh**: 1♀, Tirupathi, 22.x.2008, Yeshwanth, H. M. ex light trap (sodium vapour lamp) (NIM); 3♀, 3♂ ARS, Nellore, 14°25.790'N, 079°59.922'E, 03.iv.2009, Yeshwanth, ex light trap (sodium vapour lamp) (NIM); 2♂, ARS, Anakapalli, 17°41.855'N, 083°00.253'E, 5.iv.2009, Yeshwanth (NIM); **Goa**: 1♀, MRC. coll. **Karnataka**: 3♀, 1♂, Brahmavara, Udupi, 28.ix.2007, Naveen Kumar, ex light trap (sodium vapour lamp) (NIM); 2♀, Arabhavi, Belgaum, 12.ii.2008, Naveen Kumar, ex light trap (sodium vapour lamp) (NIM); 2♂, same data as former except 13.ii.2008, Yadava Babu; 1♀, Bandipur, Hangla, 19.xi.2008, 11°42.075'N, 076°37.685'E, Yeshwanth (NIM); 1♀, Markanja, D. Kannada, 12.xi.2009, Umeshkumar, ex light trap (sodium vapour lamp) (NIM); 1♀, Hessaraghata, 1.xii.2008, Umeshkumar (NIM); 7♂, 2♀, Brahmavara, Udupi, 13°25'N, 74°45'E, 79 m, 29.v.2014, Naveen, ex light trap (NIM); 1♀, CPCRI, Vittal, 06.iv.2018, David, K. J. (NIM); **Kerala**: 2♀, Kozhikode, 24.i.2008, 11°16.678'N, 075°50.647'E, Naveen Kumar, ex light trap (sodium vapour lamp) (NIM); 1♂, 1♀, ZSI, Calicut, 11°25'N, 75°77'E, 18.xi.2012, Devaraj, R., ex light trap (sodium vapour lamp), NIM-2022 (NIM); 1♂, CPCRI, Kasaragod, 12°31'N, 74°57'E, 6.xii.2012, Umeshkumar, S., ex light trap (sodium vapour lamp), NIM-2022 (NIM); 1♂, same data as before except date 4.xii.2012 (NIM); 2♂, 1♀, ZSI, Calicut, 24.iv.2014, V. Naveen, ex light trap (NIM); 1♀, no label (NIM); **Tamil Nadu**: 1♀, Yelagiri, Javadi Hills, 16.vi.2016, Ramesh Kumar, A.

**Figures 76–79.**
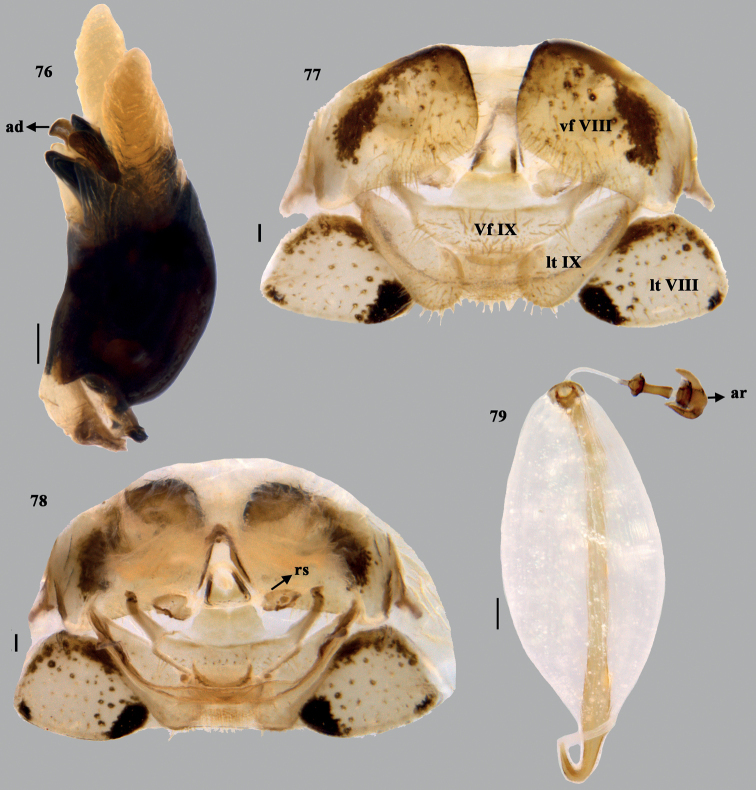
*Mycterizonbellus* (Distant), stat. rev. **76** phallus (lateral) **77** terminalia (dorsal) **78** terminalia (ventral) **79** spermatheca. Scale bar: 0.1 mm. Abbreviations: ad – aedeagus; ar – apical receptacle; rs – ring sclerites; lt VIII – laterotergite VIII; lt IX – laterotergite IX; vf VIII – valvifers VIII; vf IX – valvifers IX.

#### Redescription.

***Colouration*** (Figs 63, 64). Dorsum ochraceous with black irregular spots scattered over pronotum, scutellum and hemelytra; head ochraceous except narrow lateral margins of mandibular plates, two oblique, narrow longitudinal lines on disc of clypeus, black; antennae ochraceous; lateral margins of pronotum adjacent to humeri (including humeri), black; disc of scutellum with roughly anchor-shaped spot and one short oblique stripe each at basal angles of scutellum, black; connexivum with narrow, transverse, black stripe on anterior and posterior margin of connexivial segments, black; sometimes anterior 1/2 margin of each pleurite III–VII with longitudinal stripe, black; distal end of hemelytra with moderate size spot, ochraceous, sometimes with purplish shade; membrane smoky brown; ventral side ochraceous with the following markings: thoracic pleura laterally with irregular markings mainly formed by the coarse punctures, one roughly circular spot mesad at an oblique angle to each spiracles; short, narrow and transverse stripe below each spiracles on either side of abdominal sternites III–VII and anterior and posterior angles of each abdominal sternites III–VII, black; labium ochraceous with apex black; legs ochraceous except small nearly circular spots densely distributed on femora and tibiae (finer on tibiae) and apical 1/2 of claws, black.

***Integument and vestiture*.** Body above covered with coarse, black punctures confluent to form irregular markings on dorsum especially on pronotum and scutellum. Ventral side of body with coarse and dense black punctures mostly concentrated towards lateral sides on thoracic sternites. Femora of legs with coarse, black, dense punctures and tibiae with fine black punctures. The punctures finer on abdominal sternites, concentrated towards laterally. Ventral side of genital capsule including posterolateral lobes with fine pale brown punctures; terminalia with valvifers VIII and laterotergite VIII with sparse, coarse, brown punctures.

Body glabrous, except the following: antennae with fine, golden pubescence; legs with setae moderately elongate and semi erect; tibiae with dense, brown, short, semi erect setae on ventral side and dorsally with moderately elongate brown setae, dense mat of setae at the posterior end of tibiae; tarsomeres on dorsal side with moderately elongate golden setae and dense mat of short, golden setae on ventral side of all tarsomeres; labium with sparse, golden setae. Genital capsule (middle margin of dorsal rim, posterolateral lobes and the caudal 1/2 of genital capsule on ventral side) with moderately elongate, dense, semi erect, golden setae. Female genitalia (valvifers VIII, valvifers IX, laterotergite VIII and IX) with moderately long, golden semi-erect setae.

***Structure*.** See the generic redescription.

***Male genitalia*** (Figs 66, 68–76). Genital capsule (Figs 68–70) nearly quadrangular with posterolateral lobes (= caudal lobes) well developed and broadly rounded. Dorsal rim widely and deeply excavated concave with almost straight middle margin; infoldings of dorsal rim submedially with rounded lobe-like ingrowths (ls) towards posterior aperture. Ventral rim medially shallowly excavated and broadly concave. ***Paramere*** (Figs 71, 72). Sclerotised, crown broad, subquadrate with one moderately elongate finger-like process at distal end and another short, stout, apically rounded process forming an acute angle with the distal finger-like process, short neck and small plate-like apodeme. ***Articulatory apparatus*** (Fig. 73). Support bridge complex excavated ventrally cup-like, for which the narrow, elongate, oval capitate processes (cp) attached with short dorsal connectives. ***Phallus*.** Phallotheca longer than broad, narrowed towards proximal end, dorsal region convex and ventral margin nearly straight (in lateral view); thecal process slightly developed on mid dorsal side at the distal end of phallotheca; a pair of minute, stout, sclerotised, ventrolateral tubercles at the posterior end of the phallotheca; four pairs of conjunctival processes; two pairs on dorsal side (inner pair, dcp I and outer pair dcp II), one pair each on ventral side (vcp) and lateral side (lcp) respectively. inner pair of dorsal conjunctival processes (dcp I) longest, broad and membranous, narrowed towards distal end; outer pair (dcp II) short, narrow, sclerotised with distal apex acuminate and membranous; ventral ones (vcp) moderately elongate, sclerotised and apically rounded; lateral pairs (lcp) shortest and membranous; processes of aedeagus (pa) fused mid longitudinally and sclerotised except median longitudinal part; partially enclosing aedeagus (ad) at the proximal end; aedeagus (ad) moderately elongate, sclerotised and tube-like with oblique phallotreme.

**Figures 80–85.**
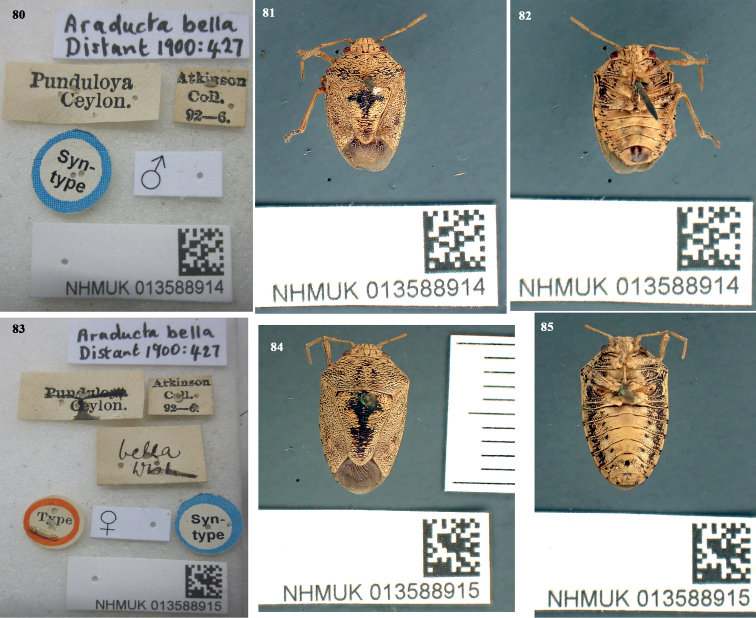
*Mycterizonbellus* (Distant), stat. rev. **80** label data of lectotype **81** lectotype (dorsal habitus) **82** lectotype (ventral habitus) **83** label data of paralectotype **84** paralectotype (dorsal habitus) **85** paralectotype (ventral habitus). Photographed by Mick Webb, NHM, London, UK.

***Female genitalia*.** (Figs 67, 77–79). Valvifers VIII (vf VIII) transverse, broad and roughly quadrangular, with medial margins nearly straight; inner posterolateral angles rounded; posterior margin nearly straight; valvifers IX (vf IX) single, transverse, broad sclerite; laterotergites IX (lt IX) oblique, elongate with inner and outer margin straight, caudal margin orbicular or rounded, caudal apex smooth, without any denticle; laterotergite VIII (lt VIII) subtriangular, caudal margin smooth, slightly convex. A pair of ring sclerites (rs) oblong. ***Spermatheca*.** Spermathecal dilation long, regularly, obliquely fluted; proximal end of sclerotised rod, at the middle of lumen of spermathecal dilation, curved upwards and continued as distal spermathecal duct; proximal flange 1/2 the size of distal flange; apical receptacle (ar) orbicular with three elongate ductules, all facing towards distal rim and surpassing distal rim.

#### Measurements

**(mm).** Males (*n* = 5) as median (minimum–maximum). Body length 8.63 (8.08–9.09); head: length 1.90 (1.82–1.98), width (including eyes) 2.91 (2.85–3.01), interocular width 1.74 (1.65–1.78); lengths of antennomeres: I – 0.56 (0.46–0.68), II – 0.68 (0.54–0.77), III – 1.25 (1.20–1.33), IV – 1.58 (1.47–1.68), V – 1.55 (1.50–1.63); lengths of labiomeres: I – 0.63 (0.51–0.73), II – 0.86 (0.79–0.93), III – 0.60 (0.56–0.63), IV – 0.52 (0.46–0.60); pronotum: length 2.12 (1.92–2.33), width (including humeri) 5.07 (4.82–5.32); scutellum: length 4.17 (3.84–4.31), width (at basal angles) 3.33 (3.28–3.48).

Females (*n* = 5) as median (minimum–maximum). Body length 10.26 (9.83–10.73); head: length 2.05 (2.01–2.13), width (including eyes) 3.19 (3.12–3.37), interocular width 1.99 (1.97–2.04); lengths of antennomeres: I –0.59 (0.55–0.64), II – 0.68 (0.56–0.74), III – 1.35 (1.26–1.44), IV – 1.72 (1.71–1.76), V –1.74 (1.73–1.75); lengths of labiomeres: I –0.61 (0.54–0.66), II –0.97 (0.91–1.04), III – 0.61 (0.56–0.75), IV – 0.53 (0.51–0.60); pronotum: length 2.63 (2.40–2.78), width (including humeri) 5.95 (5.62–6.44); scutellum: length 4.94 (4.64–5.31), width (at basal angles) 3.90 (3.70–4.11).

#### Differential diagnosis and remarks.

Though the species *bellus* neither possess the basal abdominal tubercle nor the metasternal cruciform process, Distant (1900a) placed *bellus* in *Araducta*; however, he mentioned in the redescription of *Araducta*: “…Metasternum with central, cruciform process, not notched posteriorly. Second abdominal segment with a central, broad, obtuse, spinous tubercle...” Later, Distant (1902) proposed the genus *Dunnius* and the original description of *Dunnius* again mentioned the characters of the central cruciform process and the basal abdominal tubercle, but he transferred *bellus* from *Araducta* to *Dunnius*. Breddin (1909) recognised *D.bellus* as a distinct species (see comments under *Mycterizon*) and found that it was not congeneric with *Dunnius*, hence proposed *Mycterizon* to accommodate it. However, Distant (1918) synonymised this genus with *Dunnius* and transferred *bellus* back to *Dunnius* without justifying his action. The present study is in agreement with Breddin (1909) (see comments under *Mycterizon*) and therefore *Mycterizonbellus* (Distant, 1902) is removed from synonymy with *Dunniusbellus* (Distant, 1902). The single male syntype of *Araductabella*, presently lodged in NHM, London, UK, is herewith designated as lectotype and the female syntype as paralectotype, also currently in NHM, London, UK.

#### Distribution.

India: Bihar, Odisha, Tamil Nadu (Distant 1918); Ceylon (= Sri Lanka) (Distant 1900a).

##### ﻿Tribal placement of *Mycterizon*

The genus *Dunnius* originally included four species, viz., *D.bellus*, *D.fulvescens*, *D.minor*, and *D.sordida* (Kirby) (Rider, 2022). The aforementioned nomenclatural changes and new taxa descriptions (*A.sordida* (Kirby, 1891), comb. nov; *M.bellus* (Distant, 1902), stat. rev.; *D.laticeps* (Zheng & Liu, 1987), comb. nov.; *D.tridentatus* (Xiong & Liu, 1995), comb. nov.; *D.trifasciatus* (Xiong & Liu, 1995), comb. nov.; and *Dunniusbarpetensis* Salini & Rabbani, sp. nov.) led to the addition of two more species to the genus *Dunnius*. The genus *Mycterizon*, monotypic with *M.bellus*, is designated as type species by monotypy and presently considered as a member of Menidini, although its tribal placement is dubious. Breddin (1909) opined that *Mycterizon* is closely related to *Antestia*, a member of Antestiini. The tribe Menidini is a large and variable one, the monophyly of which has not yet been proved. It was formally named by Atkinson (1888) as Menidaria and was defined based on a wide variety of characters: basal abdominal segment is obtusely convex or with a slender spine or rounded or compressed; if tuberculate, tibiae rounded; very rarely obtusely tuberculate at middle; metasternum not elevated, mesosternal ridge with uniform breadth throughout its length; labium usually extending beyond mid coxae, either reaching or extending beyond basal abdominal segment; third labial segment sometimes slightly longer than second; tibiae usually sulcate, if rounded, basal abdominal sternum with a spine, which usually does not extend beyond mid coxae.

Based on the examination of Indian Menidini and perusal of literature revealed that the members of this tribe do not appear to share a common ancestor and can be broadly assembled into three groups based on the combination of characters like shape of the scutellum, presence/absence of abdominal spine/tubercle, development of metasternal carinae:

Group 1 – spatulate scutellum, elongate abdominal spine present, metasternal carina narrow throughout its length;

Group 2 – subtriangular scutellum, elongate abdominal spine present, metasternal carina narrow throughout its length;

Group 3 – subtriangular scutellum, abdominal tubercle present, apex of which is placed in metasternal carina (broadening posteriorly).

*Mycterizonbellus* does not fit into any of the above-mentioned groups, but rather possesses a unique combination of characters such as a spatulate scutellum, a narrow metasternal carina, and the absence of either an abdominal spine or a tubercle. It also possesses other characters such as the head flat above, as broad as 1.5 times the head length, the external scent efferent system with peritreme well developed, short, nearly reaching mid-metapleuron, spout-like, obliquely elevated to the pleural surface, the shape of the genital capsule including a dorsal rim (slightly concave medially) and ventral rim (broadly concave, without denticles on infoldings), the shape of parameres (broad crown-shaped with stout, straight, finger-like process), and the phallus with elongate conjunctival processes. Considering these remarkable differences, it cannot be considered as a member of Menidini. Therefore, it is removed from the tribe and its tribal status cannot be decided except through phylogenetic studies using both morphological and molecular characters of the taxa included in Menidini and other related tribes.

## Supplementary Material

XML Treatment for Acesines

XML Treatment for Acesines bambusana

XML Treatment for Acesines breviceps

XML Treatment for Acesines sordida

XML Treatment for Dunnius

XML Treatment for Dunnius barpetensis

XML Treatment for Dunnius fulvescens

XML Treatment for Dunnius laticeps

XML Treatment for Dunnius tridentatus

XML Treatment for Dunnius trifasciatus

XML Treatment for Mycterizon

XML Treatment for Mycterizon bellus

## Supplementary materials

Supplementary material 1 Part 1 Data type Phylogeny figure (image file) https://binary.pensoft.net/file/811005This dataset is made available under the Open Database License (http://opendatacommons.org/licenses/odbl/1.0/). The Open Database License (ODbL) is a license agreement intended to allow users to freely share, modify, and use this Dataset while maintaining this same freedom for others, provided that the original source and author(s) are credited.Santhamma Salini, R. G. Gracy, Romila Akoijam, M. K. Rabbani, K. Jacob David, Marcos Roca-Cusachs

Supplementary material 2 Part 2 Data type Phylogeny figure (image file) https://binary.pensoft.net/file/811006This dataset is made available under the Open Database License (http://opendatacommons.org/licenses/odbl/1.0/). The Open Database License (ODbL) is a license agreement intended to allow users to freely share, modify, and use this Dataset while maintaining this same freedom for others, provided that the original source and author(s) are credited.Santhamma Salini, R. G. Gracy, Romila Akoijam, M. K. Rabbani, K. Jacob David, Marcos Roca-Cusachs
