# Supplementary figures and images for: ﻿Revision of Acesines Stål and Dunnius Distant, resurrection of Mycterizon Breddin (Hemiptera, Heteroptera, Pentatomidae, Pentatominae), and description of a new species from India
Source: Zookeys. 2023 Feb 16;1148:79–117. doi: 10.3897/zookeys.1148.95629 (PMC10208443; doi:10.3897/zookeys.1148.95629)

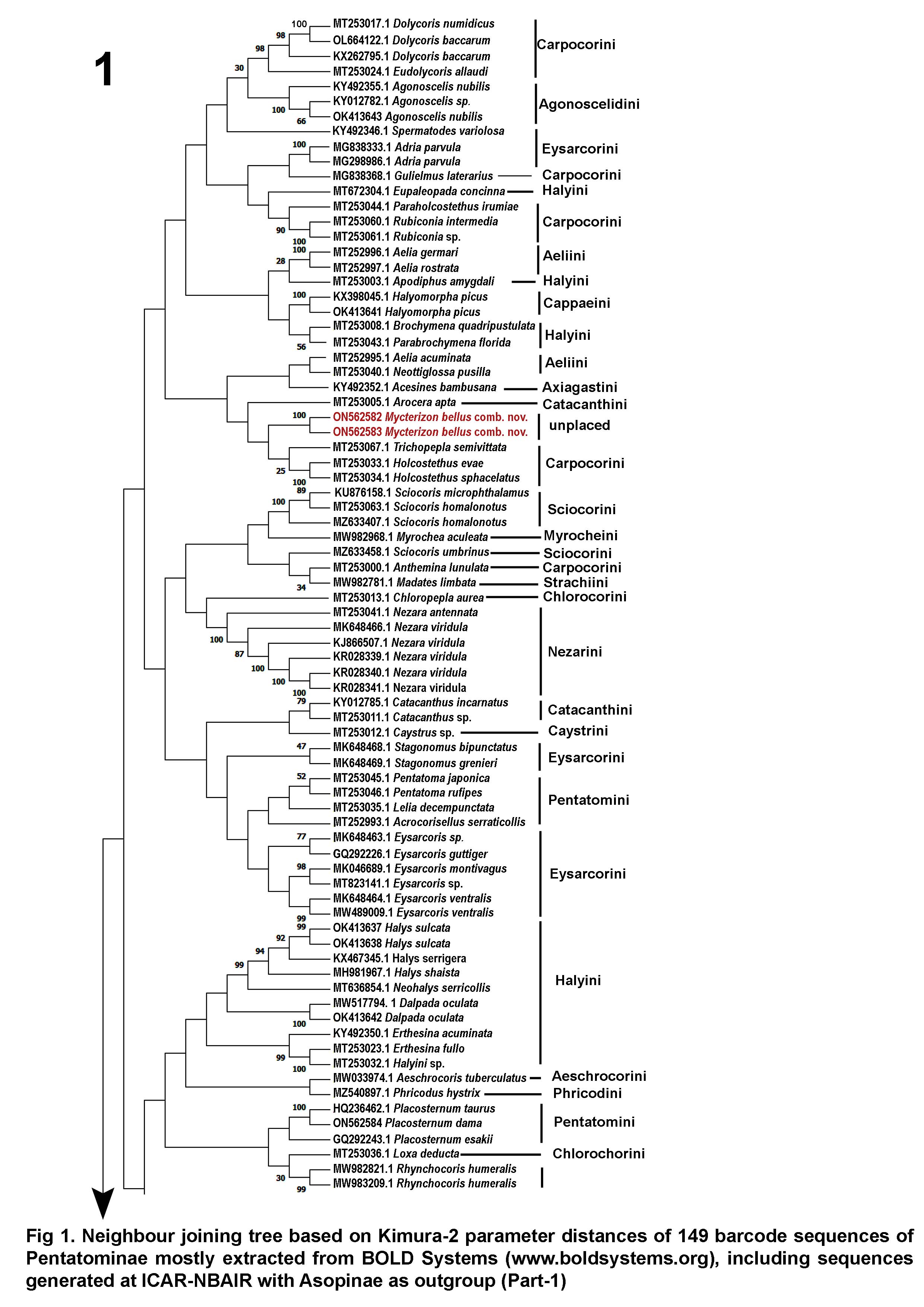

Supplement: Supplementary material 1 — Part 1 [file zookeys-1148-079_article-95629__-s001.jpg]

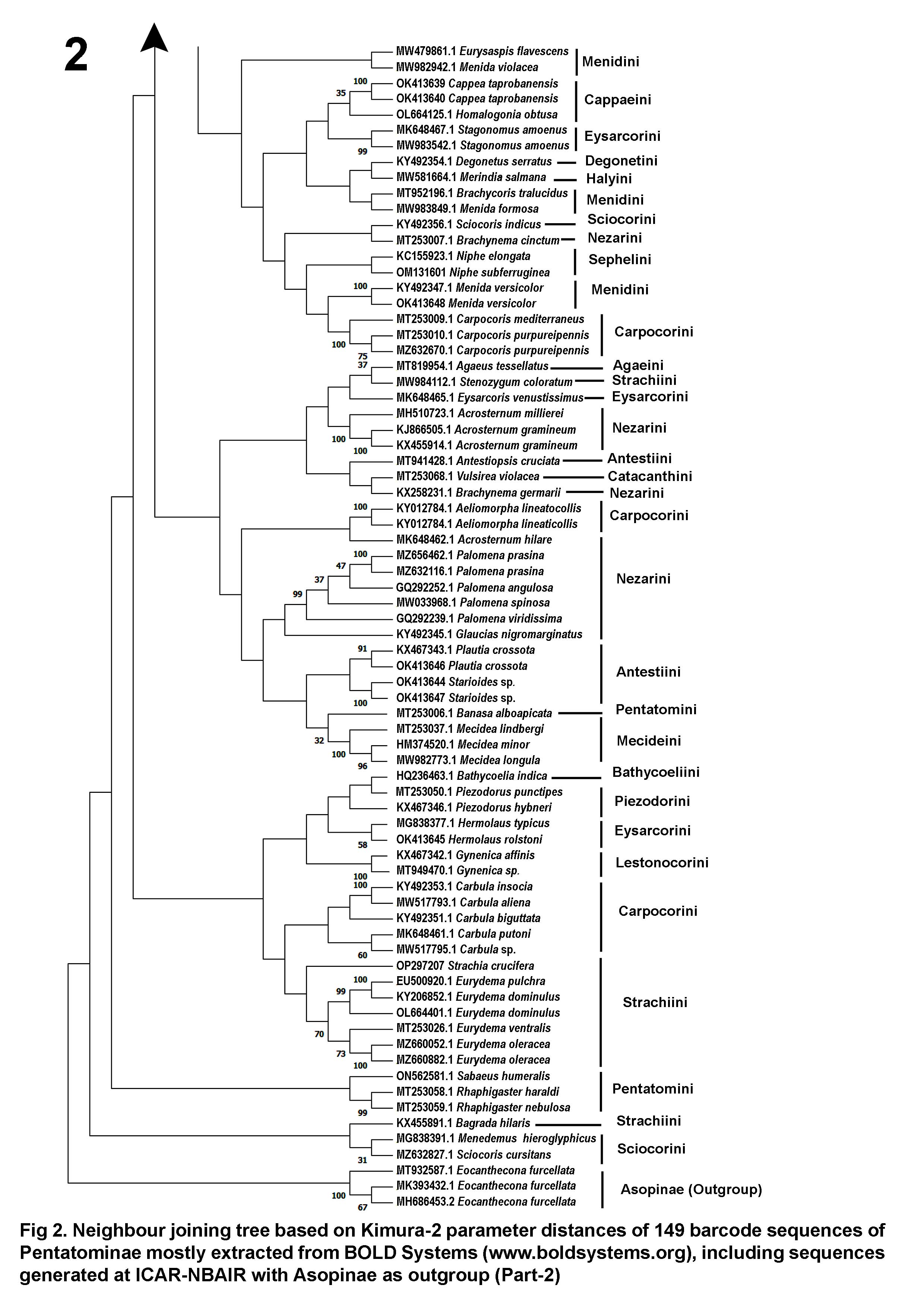

Supplement: Supplementary material 2 — Part 2 [file zookeys-1148-079_article-95629__-s002.jpg]
